# Assessment of the effects of cannabidiol and a CBD-rich hemp extract in *Caenorhabditis elegans*

**DOI:** 10.3389/ftox.2024.1469341

**Published:** 2024-10-01

**Authors:** Jessica A. Camacho, Bonnie Welch, Martine Ferguson, Estatira Sepehr, Cory Vaught, Yang Zhao, Suzanne Fitzpatrick, Jeffrey Yourick, Robert L. Sprando, Piper Reid Hunt

**Affiliations:** ^1^ Division of Food Contact Substances, Office of Food Additive Safety, Center for Food Safety and Applied Nutrition, United States Food and Drug Administration, College Park, MD, United States; ^2^ Division of Virulence Assessment, Office of Applied Research and Safety Assessment, Center for Food Safety and Applied Nutrition, United States Food and Drug Administration, College Park, MD, United States; ^3^ Biostatistics and Bioinformatics Staff, Office of Analytics and Outreach, Center for Food Safety and Applied Nutrition, United States Food and Drug Administration, College Park, MD, United States; ^4^ Division of Toxicology, Office of Applied Research and Safety Assessment, Center for Food Safety and Applied Nutrition, United States Food and Drug Administration, Laurel, MD, United States; ^5^ Office of the Center Director, Center for Food Safety and Applied Nutrition, United States Food and Drug Administration, College Park, MD, United States

**Keywords:** cannabidiol, hemp extract, *C. elegans*, alternative *in vivo* toxicity test model, irreversible developmental effect, chronic exposure effects

## Abstract

Consumer use of cannabidiol (CBD) is growing, but there are still data gaps regarding its possible adverse effects on reproduction and development. Multiple pathways and signaling cascades involved in organismal development and neuronal function, including endocannabinoid synthesis and signaling systems, are well conserved across phyla, suggesting that *Caenorhabditis elegans* can model the *in vivo* effects of exogenous cannabinoids. The effects in *C. elegans* on oxidative stress response (OxStrR), developmental timing, juvenile and adult spontaneous locomotor activity, reproductive output, and organismal CBD concentrations were assessed after exposure to purified CBD or a hemp extract suspended in 0.5% sesame oil emulsions. In *C. elegans*, this emulsion vehicle is equivalent to a high-fat diet (HFD). As in mammals, HFD was associated with oxidative-stress-related gene expression in *C. elegans* adults. CBD reduced HFD-induced OxStrR in transgenic adults and counteracted the hypoactivity observed in HFD-exposed wild-type adults. In *C. elegans* exposed to CBD from the onset of feeding, delays in later milestone acquisition were irreversible, while later juvenile locomotor activity effects were reversible after the removal of CBD exposure. CBD-induced reductions in mean juvenile population body size were cumulative when chronic exposures were initiated at parental reproductive maturity. Purified CBD was slightly more toxic than matched concentrations of CBD in hemp extract for all tested endpoints, and both were more toxic to juveniles than to adults. Dosimetry indicated that all adverse effect levels observed in *C. elegans* far exceeded recommended CBD dosages for humans.

## 1 Introduction

Endocannabinoid signaling plays an essential role in organismal development, and exposure to exogenous cannabinoids can alter the activity of these signaling pathways, resulting in adverse outcomes such as low birth weight, preterm birth, and increased need for neonatal intensive care (Campolongo et al., 2011; ACOG, 2017; Gunn et al., 2016; Baía and Domingues, 2022). Despite the growing use of cannabidiol (CBD) for stress relief, relaxation, and sleep improvement, few studies have assessed the effects of CBD exposure, either purified or in hemp plant extracts, during pregnancy and early development, thus creating critical data gaps (Lee and Hardy, 2021; Argueta et al., 2020; Wheeler et al., 2020; Saleska et al., 2022).

*Caenorhabditis elegans* are non-pathogenic small nematodes with a 3-day lifecycle. Numerous genetic pathways involved in organismal development, neuronal function, and responses to toxic chemicals are conserved across phyla, including humans and nematodes, suggesting that toxicity data from small model organisms such as *C. elegans* can contribute to weight of evidence evaluations for safety assessment (Leung et al., 2008; Masjosthusmann et al., 2018; Hartman et al., 2021; EMA/CHMP/ICH, 2020). Multiple aspects of endocannabinoid signaling are conserved from nematodes to humans (Pastuhov et al., 2016; Levichev et al., 2023). Endocannabinoid transmitters 2-arachidonoylglycerol (2-AG) and anandamide (AEA) have been found throughout the animal kingdom, and *C. elegans* encodes members of each of the seven transient receptor potential ionotropic cannabinoid receptor subfamilies (Paulsen and Burrell, 2019). Both 2-AG and AEA have indirect effects on monoaminergic (serotonin, dopamine, GABA) neurotransmitter signaling in rodents and *C. elegans* (Oakes et al., 2019; Linge et al., 2016; Huestis et al., 2019; Fernandez-Ruiz et al., 2010). Increased levels of endocannabinoids increase the latency to antinociceptive behaviors in mice (tail withdrawal in response to heat) (Long et al., 2009) and *C. elegans* (aversive responses to a noxious chemical) (Oakes et al., 2017). In *C. elegans* and mammals, AEA has analogous effects on appetitive and consummatory feeding behaviors (Levichev et al., 2023). These AEA-induced effects on feeding behavior in *C. elegans* require the mammalian CB1 receptor ortholog NPR-19, and the transgenic human CB1 receptor gene CNR1 can restore the behavioral responses to AEA in *C. elegans npr-19* null mutants (Oakes et al., 2017; Levichev et al., 2023). Taken together, these findings indicate that *C. elegans* may be an informative model for evaluating the apical *in vivo* effects of hemp-derived cannabinoids.

Within tiered testing or assay battery approaches, alternative *in vivo* models can contribute to developmental and reproductive toxicity assessments (EMA/CHMP/ICH, 2020). The *C. elegans* model has high positive predictive values for mammalian developmental toxicants, neurotoxicants, and conserved modes of chemical action (Boyd et al., 2016; Cole et al., 2004; Hartman et al., 2021; Masjosthusmann et al., 2018). In this study, the effects of purified CBD and a CBD-rich hemp extract were assessed in *C. elegans* juveniles on body size, timing of achievement of developmental milestones, and locomotor activity, and in adults on body size, oxidative stress response, spontaneous locomotor activity, and reproductive output. Whole animal tissue concentrations of CBD were also assessed after 24 h exposure in both juveniles and adults.

The *C. elegans* cuticle provides a protective and highly impervious barrier between the animal and its environment (Page and Johnstone, 2007); therefore, exposure in *C. elegans* toxicity testing is primarily via the oral route. Although absorption can occur through the cuticle and underlying ectodermal tissues, especially during larval molting, over the course of development this is likely to be a small fraction relative to exposure via the digestive tract. The micro-packaging of hydrophobic nutraceuticals and functional plant extracts in emulsions improves chemical stability and delivery, making the use of emulsions in beverages an emerging trend in the food industry (Tireki, 2021). Emulsions have been successfully used for the oral delivery of a hydrophobic nutraceutical in *C. elegans* (Shen et al., 2019). Our initial experiments using CBD sesame-oil-in-milk emulsions were found to produce dose-response effects on oxidative stress response and developmental timing, so the emulsion delivery method was used for this study.

## 2 Materials and methods

### 2.1 Reagents and test articles

Tween 80 (polysorbate 80) was obtained from Sigma-Aldrich (St. Louis, MO, United States), CAS number 9005-65-6. Sesame oil was obtained from Sigma-Aldrich, CAS number 8008-74-0, stored at 4°C and used within 3 months of opening the bottle. Purified cannabidiol (CBD) was obtained from Cayman Chemical (Ann Arbor, MI, United States), CAS number 13956-29-1, ≥98% purity. Isotopically labeled internal standard stock solution of CBD-D_3_ (SKU: C-084, 0.1 mg/mL in methanol, Cerilliant^®^) was purchased from Sigma-Aldrich. Acetonitrile (CHROMA-SOLV ™, LC-MS grade) was purchased from Fisher Scientific (Hampton, NH, United States). Ultrapure deionized water (resistivity 18.2 MΩ-cm) was produced using a Milli-Q system from Millipore Sigma (Burlington, MA, United States).

An ethanolic hemp extract was provided by the National Center for Natural Products Research at the University of Mississippi, University, MS, USA. The concentrations of cannabinoids in the extract were reported to be: tetrahydrocannabinol at 1.6 mg/mL, CBD at 52.6 mg/mL, cannabichromene at 1.5 mg/mL, tetrahydrocannabivarin at 0.1 mg/mL, cannabigerol at 1.3 mg/mL, and cannabinol at 0.2 mg/mL (Zhao et al., 2023). These concentrations were verified to be ≤7.1% difference prior to use. Ethanol was removed from the hemp extract using a CentriVap^®^ Centrifugal Concentrator from Labconco^®^ Corporation (Kansas City, MO). Two cycles of centrifugation with no heat were used to evaporate ethanol from the samples. Between each cycle, the samples were placed on ice for 10 min to cool. After centrifugation and evaporation, samples were reconstituted in sesame oil, and CBD concentration was measured by ultra-high performance liquid chromatography-electrospray tandem mass spectrometry (UHPLC-ESI MS/MS, Agilent Technologies, Santa Clara, CA) prior to the experiments. Verified concentrations were then used to calibrate hemp extract exposures by CBD concentration. Initial hemp extract resuspensions in sesame oil allowed for the maximum exposures of 960 μg/mL CBD used for the experiments described in Sections 3.2–3.4. Later preparations achieved slightly more concentrated hemp extract in sesame oil, allowing for the 1,000 μg/mL CBD exposures used for the experiments in Sections 3.5 and 3.6.

### 2.2 Strains and maintenance

*C. elegans* strains CL2166 (*gst-4*p::GFP, oxidative stress response), PD4251 (*myo-3*p::GFP, progeny-to-adult ratio assay), and wild-type (N2, all other assays) were supplied by the *Caenorhabditis* Genetics Center (CGC), which is supported by the US National Institutes of Health—Office of Research Infrastructure Programs (P40 OD010440). All strains were initially grown on agar plates with a continuous ample *E. coli* food supply prior to hypochlorite treatment and transfer to *C. elegans* habitation medium (CeHM) —a mixture of 80% chemically defined nutrient medium and 20% non-fat cows’ milk (Clegg et al., 2002; Sprando et al., 2009). CeHM was prepared as per Hunt et al. (2023). Prior to use in testing, frozen strains were maintained in CeHM in vented flasks on orbital shakers inside hot/cold incubators set to 20°C for a minimum of 3 weeks with continuous ample food supply. To avoid genetic drift, strain aliquots were utilized for a maximum of 6 months prior to acclimating a fresh aliquot that had been frozen within a few weeks of receipt from the repository. Crowded culture flasks or those with dauers were discarded. *C. elegans* males spontaneously arise only rarely and do not seem to propagate in liquid culture on shakers; therefore, exposed *C. elegans* were presumed to be hermaphrodites. Culture maintenance and exposures were performed in incubators set to 20°C unless otherwise noted.

### 2.3 Emulsion preparation, assessment, and exposure methods

Stable emulsions can be prepared by adding an emulsifier and a surfactant to a mixture of oil-in-water (Zhang et al., 2020). For the emulsions in this study, the proteins in cow’s milk acted as emulsifiers, and the Tween 80 (Polysorbate 80) as a food-grade surfactant. An example emulsion preparation worksheet is provided as Supplementary Table 2.

Step 1, mixture preparation: 1 mL of either a) sesame oil, b) purified CBD in sesame oil, or c) hemp extract of predetermined CBD concentration in sesame oil was mixed with 19 mL of a mixture of 1% Tween 80 in non-fat cows’ milk (1%Tw80).
Step 2, emulsion preparation: each mixture was vortexed for at least 1 min, then passed eight times through an LV1 microfluidizer (Microfluidics, Westwood, MA, Unites States) at 10 kpsi.
Step 3, exposure: emulsions were then used as 10× dosing solutions in CeHM for final exposures of 0.5% sesame oil and 0.095% Tw80. Early evaluations comparing 10× dosing solutions of water, non-fat cows’ milk, and 1%Tw80 did not produce differences in oxidative stress response or developmental timing (data not shown). Hence, subsequent experiments used only two negative controls: the vehicle control emulsion (VCe) and 1%Tw80 for dosing.

Emulsion particle size and zeta potential were measured with a ZetaPALS from Brookhaven Instruments (Holtsville, New York) using 92-nm latex particles and BI-ZR5 Zeta potential validation material (ζ = −44 ± 8 mV) from the same company as internal standards. The emulsions were utilized in such a way that experiments were completed within 8 days of emulsion preparation. No changes, such as an oily surface or precipitation in the media, were noted by eye or microscopy in exposed wells during the experiments or in the 10× dosing emulsions, which were maintained for up to 2 months at 4°C to assess stability. These observations are in contrast with initial emulsion preparations using lower psi or fewer passes through the microfluidizer, which resulted in a visible oily sheen at the top of the experimental wells within a day or two.

### 2.4 Evaluation of oxidative stress response

The transgenic *C. elegans* strain CL2166 (*gst-4*p::GFP) was used as a biomarker for the conserved Nrf2-mediated oxidative stress response. Synchronized cohorts were exposed for 24 h, from the onset of the young adult stage, as determined by at least 50% of the cohort having at least one visible primary oocyte but no internal fertilized eggs. This occurred at approximately 65 h post L1 feeding in both wild-type and CL2166 worms grown at 20°C in CeHM. The effects on *C. elegans* transgene expression, size, and optical density were evaluated with a COPAS^®^ (Union Biometrica, Holliston, MA). Because the progeny can be easily gated separately from the original exposed cohort, no contraceptive was needed. Morphometric time-of-flight (TOF) measurements can be used as phenotypic comparisons to controls to define population health. Populations with mean TOF values that differed from matched controls by > 20% were excluded from analyses. The data shown are the mean green fluorescence values relative to the control and include no effect levels where available up to the highest concentration obtainable that maintained TOF within 80% of the control. Significance was determined from a minimum of four independent experiments per exposure group for Student’s T-test *p*-values *<0.05, # <0.005. Mean changes considered biologically meaningful were ≥10% for fluorescence and ≥5% for TOF.

### 2.5 The worm Adult Activity Test (wAAT)

See Hunt et al. (2023) for detailed methods for this assay. Briefly, synchronized N2 wild-type *C. elegans* were exposed to the contraceptive 5-fluoro-2′-deoxyuridine (FUdR) when at least 50% of the cohort had reached the mid-L4 stage. Adults were exposed the following day to purified CBD or CBD-rich hemp extract. Spontaneous locomotor activity levels were recorded for 18 h with a WMicroTracker™ (Phylumtech, Santa Fe, Argentina), an activity tracking instrument placed in a hot/cold incubator set to 20°C. Data were analyzed using the web tool https://waat.galaxytrakr.org. The data presented comprise five independently generated cohorts assessed with three or four independently produced emulsion preparations.

### 2.6 Progeny ratio assay

See Camacho et al. (2023) for detailed methods for this assay. Briefly, *C. elegans* strain PD4251 grows with wild-type parameters but glows a bright green from just before hatching and throughout adulthood, facilitating the gating of small progeny from the background with a COPAS. Stock cultures and exposed cohorts were maintained at 19°C so that all experimental steps could take place between 8 am and 8 pm Age-synchronized cohorts were continuously exposed via their nutrient media from the onset of reproductive maturity (the young adult stage), as determined by microscopy, when at least half of the population had a visible primary oocyte but no fertilized eggs. An example worksheet for this assay is provided as Supplementary Table 1. Replicate plates were evaluated by COPAS at 2, 3, and 4 days post-dosing. Significance was determined from a minimum of three independent experiments per exposure group for Student’s T-test *p*-values <0.05. TOF changes of <5% were within the variance between controls for this instrument and therefore were not considered biologically meaningful.

### 2.7 The worm Development and Activity Test (wDAT)

The wDAT uses wild-type N2 *C. elegans* to measure the timing of developmental milestone acquisition and stage-specific spontaneous locomotor activity. Age-synchronized cohorts were exposed from the onset of feeding after hatching and were tracked for 3 days through the four developmental stages in 12-well plates placed in wMicroTrackers. In some wDAT experiments, cohorts were exposed continuously for 3 days. In others, as indicated, the reversibility of the developmental effect was evaluated, with *C. elegans* exposed for 24.5 h +/-0.5 h from the onset of the initial L1 feeding. These are referred to in the text as “early-only” exposures. To avoid starvation during the washing steps, worms were washed twice, two wells at a time, followed by resuspension in CeHM within 10 min of commencing the washing steps. An example worksheet for this assay is provided as Supplementary Table 1.

For this study, L1 stage-specific locomotor activity peak times and heights were evaluated differently than previously described. When peak shapes are relatively uniform across exposures, as was the case for the L2, L3, and L4 values presented here, a mean of 3.5 h of wMT data is usually sufficient to smooth activity curves and determine peaks (Camacho et al., 2022). However, the L1 activity curves for higher concentrations of CBD and hemp extract were very different in shape from the control curves. Therefore, in order to accurately reflect the time and activity at the mid-L1 stage, the peak time for all wDAT exposures was reported as half of the time to the L1–L2 lethargus (determined as the lowest wMT value between the L1 and L2 stages), and the L1 peak height was measured as the mean of 7.5 h of wMT data centered evenly around this midpoint.

At the higher concentrations of CBD, there was some loss of synchronous developmental timing, which resulted in less clear drops in wDAT activity between developmental stages in some experiments, especially in the L4-adult lethargus. Data points where some peak times had to be estimated from the overall shape of the activity curve, rather than from a clear post-peak drop in activity as seen with lethargus in synchronized populations, are indicated with a “!” in the figures. Additionally, some experiments did not meet the acceptance criteria, either for the shape of the control activity curves and/or for the observed contamination. This may be due to the microfluidizer being too large to fit into a biological safety cabinet and the 3-day duration of the wDAT providing time for contaminants to grow. The number of experiments on which the results are based is indicated in the caption for each figure.

### 2.8 CBD tissue concentration assessment

N2 *C. elegans* were exposed for 24 h at 20°C in 12-well plates with 900 µL CeHM plus 100 µL of 10× dosing emulsion per well, using either approximately 900 adults or 2,700 juveniles per well. L1 worms were counted right after the initial feeding, using a minimum of ten 5 µL samples; however, final worm numbers are estimates by necessity (Scanlan et al., 2018). Adults were exposed when at least 50% of the population had a visible internal oocyte, indicating that they had reached the young adult stage. Juveniles were exposed right after L1 feeding and counting. Adult plates were exposed on shakers to mimic the conditions used in the transgene and progeny ratio assay experiments. Juvenile plates were exposed without shaking, consistent with no shaking in the wDAT experiments. After 24 h, worm pellets were washed once each in 14 mL 0.1% Tween 80 in M9, 0.1% Tween 80 in water, and finally water. A total of 900µL of acetonitrile was added to the washed worm pellets in ∼100 µL of remaining water supernatant. To disrupt the cuticles, 2 mL Precellys hard tissue MK28-R tubes prefilled with 2.8 mm stainless steel grinding beads were used in a Precellys homogenizer (Bertin Corp., Rockville MD) set to 8500 rpm. Fifteen 30-s cycles were required to fully break up juvenile cuticles, but ten cycles were sufficient for adults. A 2-min rest on ice between homogenization cycles was used to cool the tubes. Mixed with 800 µL of acetonitrile were 100µL of worm homogenates, which were vortexed at 3,000 rpm for 30 s and then centrifuged for 10 min at 12,000 rpm to precipitate protein. Prior to analysis, 50 µL of the sample plus 50 µL of the internal standard were transferred to liquid chromatography amber glass vials with an integrated micro-insert for UHPLC-ESI-MS/MS analysis.

### 2.9 Experimental overview

**FIGURE 1:**
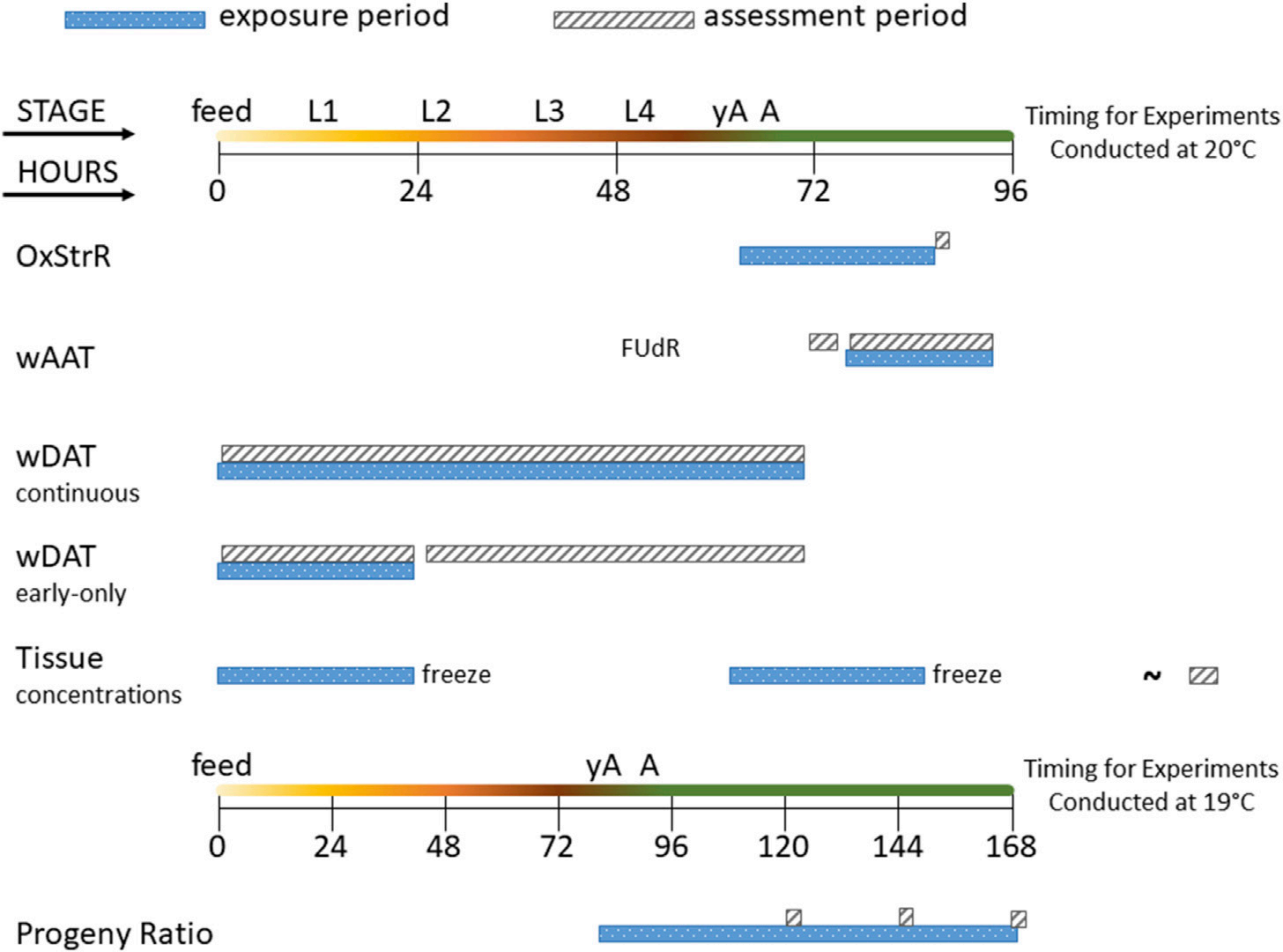
Diagram of experimental exposure and assessment periods. The time course of *C. elegans* life stages is shown from the first feeding after hatching (feed) through the four larval stages (L1–L4), young adult (yA, defined as producing oocytes but not yet having fertilized eggs in the uterus), and adult (A, at least one internal fertilized egg) stages. The oxidative stress response (OxStrR) was assessed using CL2166 *C. elegans* exposed for 24 h from the young adult stage, followed by assessment of fluorescence (a measure of gene expression) and time-of-flight (TOF, a measure of size). The worm Adult Activity Test (wAAT) assessed contraceptive (FUdR)-exposed adult wild-type *C. elegans* for spontaneous locomotor activity changes. The wAAT assay included a 2-h baseline acquisition prior to exposure, followed by exposure and tracking for 18 h. The worm Development and Activity Test (wDAT) tracked the timing and spontaneous locomotor activity of wild-type *C. elegans*. For continuous wDAT exposure, synchronous cohorts are exposed within 30 min of first feeding through to the adult stage. For early-only wDAT exposures, cohorts were exposed for the first 24 h following the first feeding, then washed, re-fed, and put back into the tracker for assessment through to the adult stage. For whole-worm tissue concentration assessments, 24 h exposures were begun either at first feeding or at the yA stage, using wild-type *C. elegans*. After exposure, worms were washed, homogenized, extracted, and frozen for later (∼) analysis. The developmental timing of *C. elegans* depends on temperature. Progeny ratio experiments were conducted using PD4251 *C. elegans* incubated at 19°C so that all experimental steps could be accomplished during the daytime. Progeny ratio exposures were continuous, beginning from the young adult stage, and replicate plates were used for analyses on days 2, 3, and 4 post-dosing.

An overview of the life stage, exposure duration, and assessment period for each of the experiments in this study is provided in Figure 1.

## 3 Results

### 3.1 Emulsions

Emulsions were prepared as described in Section 2.3 to produce 10× dosing solutions that comprised 5% sesame oil in a mixture of 1% Tween 80 in homogenized non-fat cows’ milk (1%Tw80). We mixed 20µL of vehicle control emulsion (VCe), purified CBD emulsion, or hemp extract emulsion with 1,980 µL water for zeta potential and particle size assessments.

**FIGURE 2:**
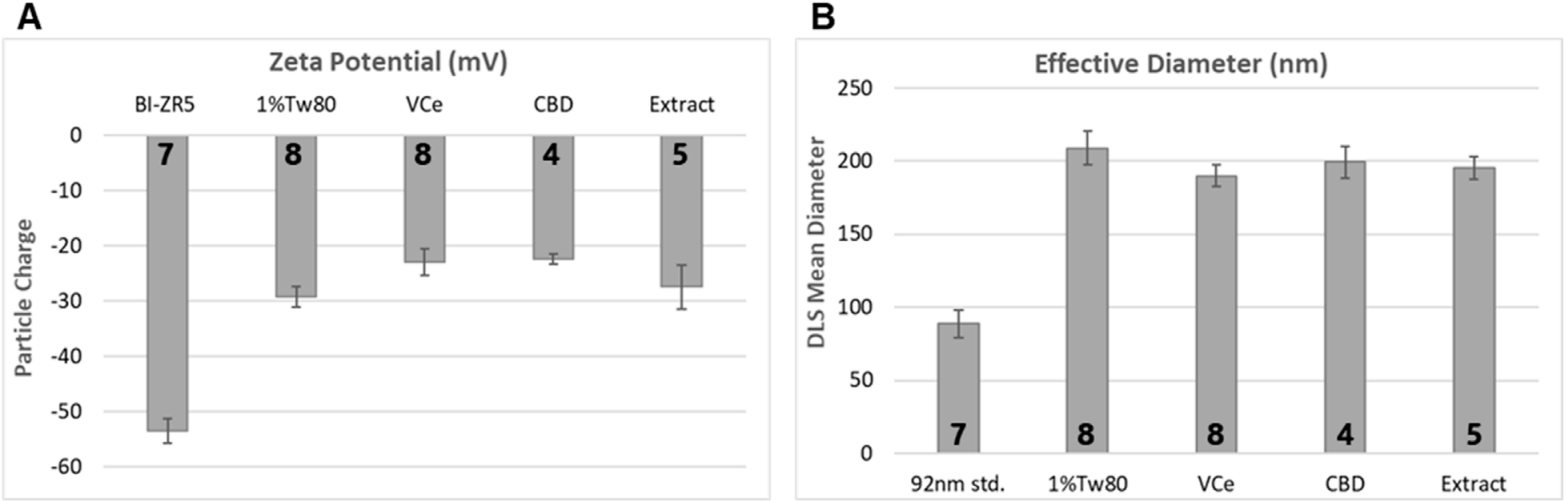
Emulsion particle charge and diameter measurements. 10× dosing emulsions were prepared using 5% sesame oil in a blend of 1% Tween 80 in non-fat cow’s milk (1%Tw80). **(A)** Mean emulsion particle charge, using BI-ZR5 as an internal reference for negatively charged particles. **(B)** Mean particle size, using 92-nm latex beads as an internal reference (92 nm std.). The number of independent experiments is listed in bold at the base of each column. Error bars indicate the standard deviation over the number of assessments listed.

Zeta potential analysis detected mean electrostatic charge on emulsion particles for 1%Tw80, VCe, CBD, and hemp extract (Extract) ranging from −20 to −30 mV (Figure 2A). BI-ZR5, an internal control for negatively charged particles, registered within the reference range. Dynamic light scattering indicated that the mean size of particles in the 1%Tw80 control was approximately 200 nm, and the emulsified oil mixtures did not differ significantly from this value (Figure 2B). Since CeHM is 20% non-fat cow’s milk, these data are consistent with the *C. elegans* responding to the emulsion particles in the same way as to their typical food source.

### 3.2 Evaluation of oxidative stress response bioreporter expression

**FIGURE 3:**
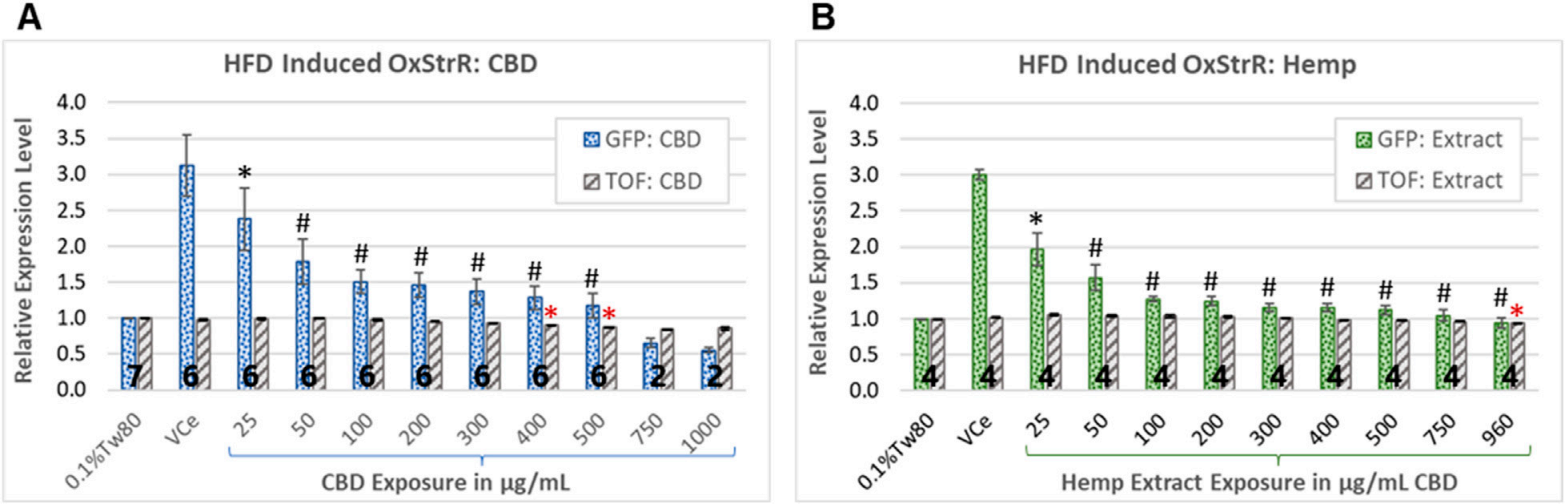
Oxidative stress bioreporter (OxStrR) gene expression and biometric analysis. GFP fluorescence and time-of-flight (TOF, a measure of body size) were measured with a COPAS microfluidic laser instrument using the transgenic *C. elegans* strain CL2166, a bioreporter for changes in oxidative stress response gene expression (OxStrR, dotted bars). **(A, B)** CBD, purified or in hemp extract, reduced OxStrR in a dose-dependent manner. **(A)** Exposure to purified CBD for 24 h from the onset of adulthood significantly reduced TOF (striped bars) at exposures of 400 μg/mL and above. **(B)** Hemp extract at matched CBD concentrations significantly reduced TOF only at 960 μg/mL. The number of independent experiments is listed in bold at the base of each column, and the error bars indicate the standard deviation among the listed number of experiments (note that the variance for TOF was very small, making the error bars appear as a single line). Bars indicate changes normalized in each experiment to the 0.1%Tw80 control. Student’s t-test *p*-values from a minimum of four independent experiments of (*) ≤ 0.05 and (#) ≤ 0.005 were considered statistically meaningful. Minimum mean changes of 5% or 10% from control were considered biologically meaningful for TOF and fluorescence, respectively. Symbols in black indicate significant differences from the VCe control for GFP bioreporter expression, while red asterisks indicate significant differences from the 0.1%Tw80 control for TOF.

Diets high in fat are associated with oxidative stress in mice, rats, and humans (Le Lay et al., 2014; Ding et al., 2023; Noeman et al., 2011; Tan and Norhaizan, 2019). Expression from the promoter of the *C. elegans glutathione S-transferase 4* (*gst-4*) gene, attached to the coding sequence for green fluorescent protein (GFP) in the transgenic strain CL2166 is used as a bioreporter of oxidative stress response (OxStrR) (Link and Johnson, 2002; Blackwell et al., 2015). To test for OxStrR, CL2166 worms were exposed from the beginning of the young adult stage, which began approximately 65 h post initial feeding after hatching. The vehicle control nanoemulsion (VCe) exposed the *C. elegans* to 0.5% sesame oil, a high-fat diet for this model. With exposure to VCe for 24 h, OxStrR transgene expression was increased three-fold over the matched oil-free vehicle control exposure of 0.1% Tween 80 in cows’ milk (0.1%Tw80) (Figures 3A,B).

CBD has been shown to reduce markers of inflammatory and oxidative processes in human cell cultures and laboratory mammals (Atalay et al., 2019). In *C. elegans* exposed to purified CBD or hemp extract for 24 h from the onset of adulthood, OxStrR gene expression was dose-dependently reduced (Figure 3, dotted bars). Compared to the VCe control, purified CBD reduced the HFD-induced OxStrR response to approximately 50% over the oil-free 0.1%Tw80 control at 100 μg/mL (Figure 3A), while an equivalent concentration of CBD in hemp extract similarly reduced reporter expression at 50 μg/mL (Figure 3B). Hemp extract at 200 μg/mL CBD and above reduced HFD-induced reporter expression to within 25% of the oil-free control, while this occurred for purified CBD only at 500 μg/mL and above.

In microfluidic laser instrument measurements, time-of-flight (TOF) is an indication of body size. Purified CBD significantly reduced TOF (Figure 3A, gray striped bars) at exposures of 400 μg/mL and above, consistent with acute toxicity at high CBD concentrations. Although purified CBD reduced OxStrR biomarker expression to below the 0.1%Tw80 oil-free control at 750 μg/mL and above, this reduction in transcription may be due to toxicity, as indicated by reduced TOF, rather than reduced oxidative stress. In contrast, hemp extract significantly reduced TOF only at the highest tested concentration. Note that in this set of experiments, the highest achieved CBD concentration in hemp extract was 960 μg/mL, not 1,000 μg/mL as with purified CBD.

### 3.3 The worm Adult Activity Test (wAAT)

Wild-type adult *C. elegans* were assessed for acute effects on spontaneous locomotor activity with exposure to emulsified CBD or hemp extract containing matched concentrations of CBD. The data presented represent experiments with five independently generated cohorts, using three independent emulsion dosing preparations. One of the five experiments from the hemp extract dataset was excluded due to not meeting all wAAT acceptance criteria for negative controls as described in Hunt et al. (2023).

**FIGURE 4:**
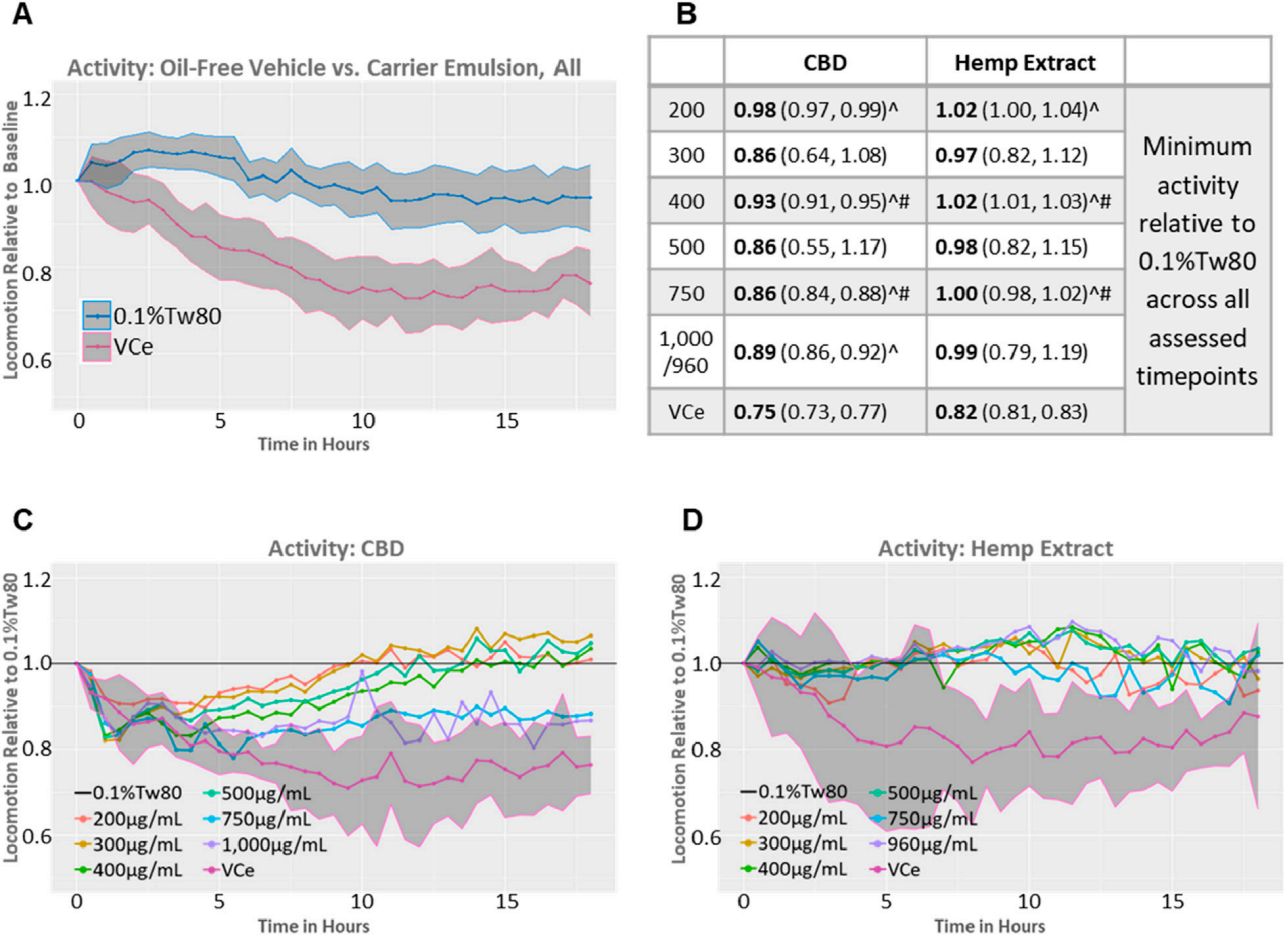
Spontaneous locomotor activity in synchronized adult populations. Mean population locomotor activity in wild-type adult *C. elegans* was measured with an infrared beam interruption device and analyzed with the web tool https://waat.galaxytrakr.org. **(A)** Graph of the mean of all oil-free control (0.1%Tw80, upper blue line) and vehicle control emulsion (VCe, lower pink line) activity levels over the course of the 18-h test for both CBD and hemp extract data sets, and the shaded areas indicate the 95% confidence band around the mean values. **(B)** Data over the 18-h test period using the model-predicted minimum activity as the determinant profiler is shown, with the model-predicted minimum value in bold and the confidence interval in parentheses. Statistically significant minimum activity greater than VCe is indicated by a ^ symbol, while # indicates concentrations where purified CBD was significantly different from CBD in hemp extracts. **(C)** Graph of the mean effects of purified CBD on activity levels relative to 0.1%Tw80. **(D)** Graph of the mean effects of CBD in hemp extract effects on activity levels relative to 0.1%Tw80.

In rodents, high-fat diets can reduce locomotor activity (Arika et al., 2019; Han et al., 2020). A comparison of the 0.5% sesame oil vehicle control emulsion (VCe) *versus* the oil-free (0.1%Tw80) controls from both datasets relative to their well-matched baseline activity prior to dosing demonstrates that VCe significantly reduced motor activity by a mean of 19% relative to 0.1%Tw80 across the 18-h test period and that the 95% confidence intervals around the means no longer overlap after 2 h of exposure (Figure 4A).

In some, but not all, rodent studies, oral CBD exposure can produce mild hyperlocomotion (Britch et al., 2017; Hlozek et al., 2017). Significant increases in locomotor activity above VCe were detected after exposures to 200, 400, and 750 μg/mL CBD, either purified or in hemp extract (Figure 4B, ^ symbol), consistent with CBD’s amelioration of the locomotion effects of a high-fat diet. Other concentrations also increased activity above VCe, but the variability (as indicated by the 95% confidence intervals shown in parentheses in Figure 4B) was too large for significance. Populations exposed to 400 or 750 μg/mL CBD from either the purified chemical or in hemp extract significantly differed from each other (Figure 4B, # symbol), with the hemp extract closer to the oil-free control. Purified CBD at 200 μg/mL to 500 μg/mL restored spontaneous locomotor activity to 0.1%Tw80 levels within approximately 8–12 h of exposure (Figure 4C), while none of the hemp exposures tested were significantly different from the oil-free 0.1%Tw80 control over the 18 h assessment period (Figures 4B,D). Together, these data indicate that purified CBD was less effective than CBD in hemp extract in counteracting the hypoactivity effects of the sesame oil emulsion in adult *C. elegans*.

Purified CBD at 750 μg/mL and 1,000 μg/mL also increased motor activity above VCe but to a lesser extent than lower concentrations of purified CBD or matched concentrations of CBD in hemp extract (Figures 4B,C). These data are consistent with a U-shaped activity curve and a reduction in benefit at 750 μg/mL and above for purified CBD. Acute toxicity, as measured by a reduction in motor activity to less than 60% of control, as previously observed with mercury and arsenic (Hunt et al., 2023), did not occur at any tested CBD concentration in either preparation.

### 3.4 Progeny ratio assay

The *C. elegans* progeny-to-adult ratio assay measures the number of progeny relative to the number of adults in control and exposed populations, and it also provides information on microfluidic/laser technology morphometry as time-of-flight (TOF), a measure of size. Age-synchronized *C. elegans* were continuously exposed from the onset of reproductive maturity in the parental cohort. Replicate plates were assessed at 2-, 3-, and 4-days post-dosing, equivalent to days 5–7 post L1 feeding (pL1f) of the parental population. Initial testing was also conducted on parental day 8 pL1f, but the control parental and progeny populations could no longer be separated by gating at that point.

**FIGURE 5:**
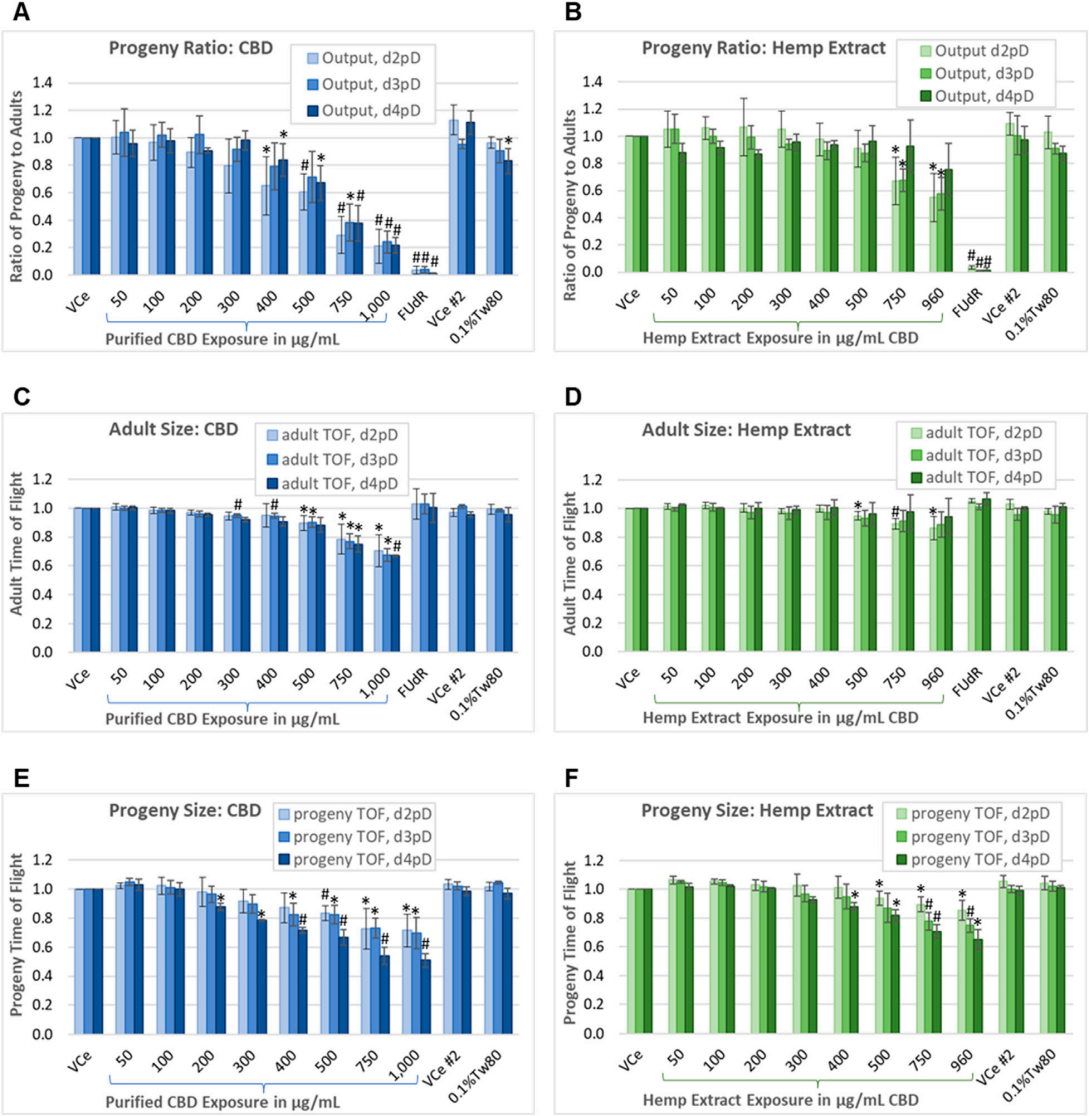
Progeny-to-adult ratio and biometric analysis. Assessment on days 2, 3, and 4 post-dosing of the ratio of progeny to adults plus morphometry in populations continuously exposed from the onset of reproductive maturity in the parental population. This assay uses PD4251, a *C. elegans* strain that glows bright green upon laser excitation, to allow for the separation of the smallest progeny from the background noise generated by the nutrient media. **(A, B)** Ratio of progeny to adults for purified CBD, and for hemp extract concentrations matched for CBD content. **(C, D)** Mean adult population time-of-flight (TOF, an indicator of size). **(E, F)** Mean TOF of the progeny population. Changes were considered meaningful if the mean difference (three independent experiments minimum) from vehicle emulsion (VCe) control was ≥5% and values normalized to the plate-matched controls had Student’s t-test *p*-values of <0.05 (*) or <0.005 (#).

For populations exposed to purified CBD at 400 μg/mL and above, progeny output relative to the matched vehicle control emulsion (VCe) was significantly reduced in a dose-dependent manner, and these reductions were relatively consistent over the 3-day assessment period (Figure 5A). However, for the matched CBD concentrations in the hemp extract, only 750 μg/mL and 960 μg/mL significantly reduced progeny output, and only on days 2 and 3 post-dosing (d2pD and d3pD) (Figure 5B). Based on the reproductive effects of individual concentrations of purified CBD and hemp extract normalized to CBD concentration, there was a marked difference between the two, especially at the three highest tested concentrations. For example, with exposures of 750 μg/mL purified CBD, the ratio of progeny to adults was reduced by 71% on d2pD, and 62% on d3pD and d4pD relative to VCe controls. At 750 μg/mL CBD in hemp extract, the ratio of progeny to adults was reduced by 32%–33% on d2pD and d3pD, and a non-significant mean downward trend of only 7% on d4pD.

Each independent experiment for CBD or hemp extract was performed with three replicates (one for each day of assessment) of two plates, one for low concentrations and the other for high concentrations plus the oil-free 0.1%Tw80 and 5-fluoro-2′-deoxyuridine (FUdR) controls, with each plate having a VCe control. The difference in progeny output between the VCe controls on the two plates was not significantly different at any assessed time point. Progeny output for the 0.1%Tw80 control was reduced by a mean of 17% only for the CBD set of experiments, and only for d4pD. This may have been due to slight overcrowding of *C. elegans* by the fourth day of continuous exposure and consequent reduction in nutrient resources. FUdR at 5 μg/mL was used as a positive control for reducing reproductive output. In FUdR-exposed populations, few or no progeny were observed by microscopy or counted by the COPAS (Figures 5A,B). In contrast to FUdR’s effects on reproduction, adults exposed to FUdR had normal body size as measured by TOF (Figures 5C,D).

On d2pD, purified CBD was associated with mean adult body size reductions of 10%, 22%, and 30% at 500 μg/mL, 750 μg/mL, and 1,000 μg/mL, respectively (Figure 5C). On d3pD, smaller mean changes in adult body size became statistically significant at lower concentrations, with 5% reductions in TOF at 300 μg/mL and 400 μg/mL (Figure 5C); however, the overall dose-dependent trend did not change for purified CBD over the 3 days of assessment. In contrast, CBD in hemp extract only reduced adult body size by 5%, 11%, and 14% at 500 μg/mL, 750 μg/mL, and 960 μg/mL CBD, respectively, and only on d2pD (Figure 5D). This more modest reduction in adult body size, and only at the earlier stage of chronic hemp extract exposure, is consistent both with a) progeny ratio data showing recovery of progeny output by d4pD (Figure 5B) and with b) significant reductions in adult body size in the oxidative stress reporter strain only at the highest 960 μg/mL CBD in hemp extract exposure (Figure 3B).

The mean juvenile population body size was reduced dose-dependently both with purified CBD and with matched concentrations of CBD in hemp extract, although again the lowest observed adverse effect levels (LOAELs) were lower for purified CBD relative to CBD in hemp extract (Figures 5E,F). Additionally, and in contrast to adults, there was a significant downward shift in mean juvenile body size with continued population exposure, suggesting possible germ cell-mediated developmental toxicity in later progeny and/or embryonic effects. By d4pD, the mean juvenile population body size LOAEL was 200 μg/mL for purified CBD (Figure 5E) or 400 μg/mL for CBD in hemp extract (Figure 5F), with reductions of 12% for both. At 750 μg/mL continuous purified CBD exposure, mean juvenile TOF values were reduced by 27% on 2dpD and d3pD, and by 46% on d4pD. At 750 μg/mL CBD in hemp extract, mean juvenile TOF values were reduced by 11%, 22%, and 29% on days 2, 3, and 4 post-dosing, respectively. These data are consistent with the cumulative effects on progeny with continuous chronic exposure from the time of parental reproductive maturity.

Under control conditions, *C. elegans* hermaphrodites are reproductively active for approximately 6 days after reaching the adult stage (Johnson, 1987). With testing through 4 days of continuous exposure beginning at the onset of reproductive maturity, the progeny-ratio-assay-identified *C. elegans* No Observed Adverse Effect Levels (NOAELs) for purified CBD to be 300 μg/mL for reproductive output, 200 μg/mL for adult toxicity as measured by reduced mean body size, and 100 μg/mL for juvenile toxicity as measured by reduced mean body size. NOAELs identified for CBD in hemp extract were 500 μg/mL for reproductive output, 400 μg/mL for adult toxicity, and 300 μg/mL for juvenile toxicity.

### 3.5 The worm Development and Activity Test (wDAT)

**FIGURE 6:**
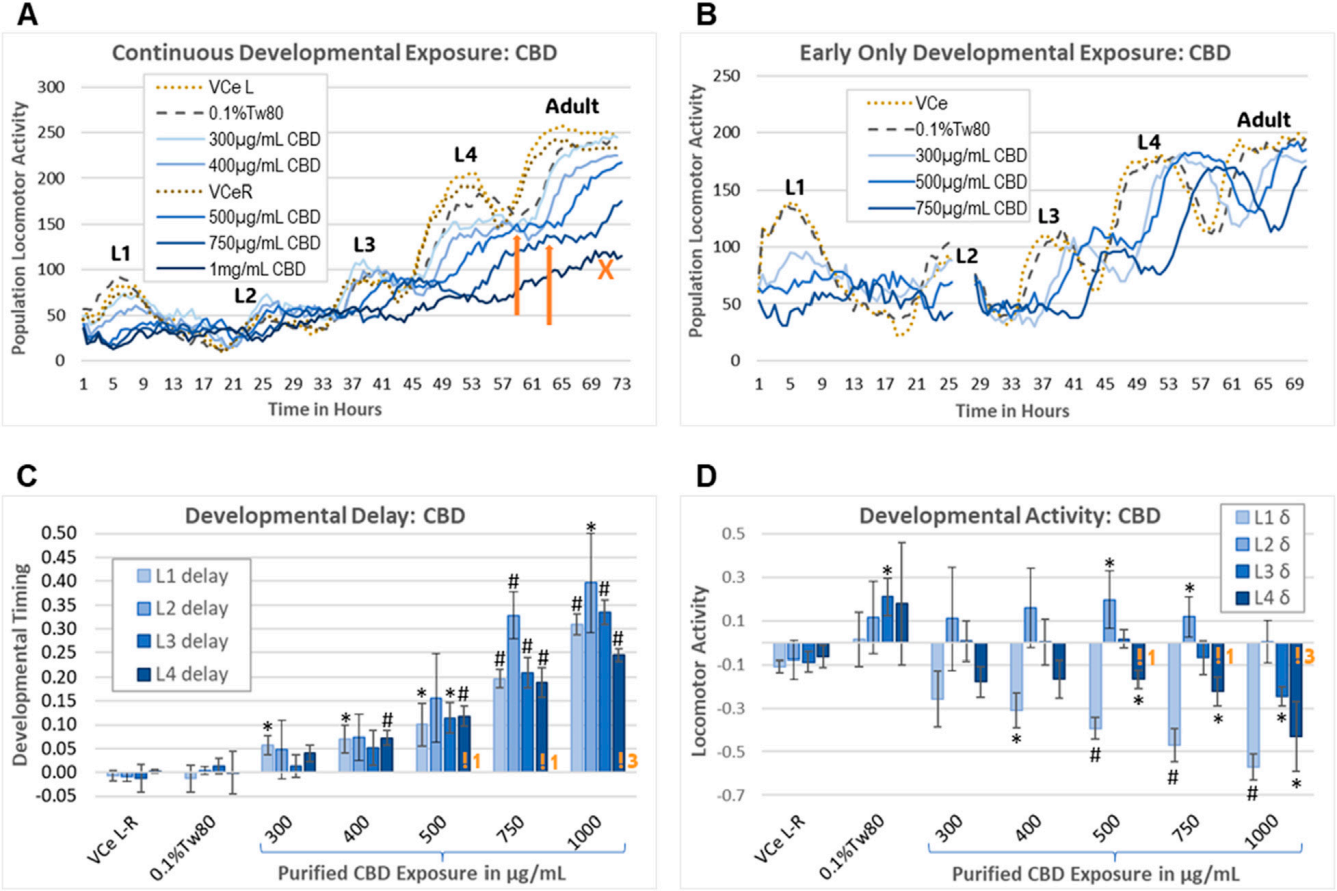
Worm Development and Activity Test (wDAT). **(A)** A single continuous exposure wDAT experiment is graphed. Activity peaks for the four larval stages are marked L1–4. “VCe L” and “VCe R” refer to the mean activity in vehicle control emulsion wells in simultaneously run left and right plates, respectively. Orange arrows indicate estimated peak times and heights. The orange “X” indicates a high concentration curve for which development within the population was not sufficiently synchronized to estimate an L4 peak. **(B)** A single early-only exposure wDAT experiment is graphed. The washing and re-feeding period is indicated by a break in the data at 25 h. Developmental delays, as indicated by a right shift of the peaks, remained after CBD was removed, but the hypoactivity, as indicated by reduced peak height in **(A)**, was not observed. **(C, D)** Data from a set of four independent continuous exposure CBD experiments are graphed. “VCe L-R” indicates the difference between control wells in plates run side-by-side, indicating experimental variability. Orange “!” symbols followed by a number indicating how many experiments for which L4 peak time needed to be estimated, consistent with loss of synchronous development at higher concentrations of CBD. For each experiment, mean values across three wells were normalized to the plate-matched vehicle emulsion (VCe) control wells. Changes were considered biologically meaningful if the mean difference from the vehicle emulsion (VCe) control was ≥5% for delay and ≥10% for activity. Significant differences in Student’s t-test *p*-values of <0.05 or <0.005 are indicated by * or #, respectively.

The wDAT uses an infrared beam locomotion tracking device to monitor both developmental timing and stage-specific locomotor activity in wells containing approximately 900 worms each, with three replicate wells per plate. *C. elegans* develop through four larval stages, designated “L1–4”, which can be monitored with the motion detection equipment due to lethargus, the brief period of reduced activity during cuticle molting between developmental stages (Figure 6A). This can be used to assess chemical effects on growth and behavior and also gives an indication of the synchrony of developmental timing within the population. The lack of decline in population locomotion after an activity peak is consistent with the microscopically observed asynchronous maturity in an exposed cohort. Peak time and height can be estimated for minor losses in synchronous cohort development, as indicated by the orange arrows pointing to the estimated L4 activity peaks for 500 μg/mL and 750 μg/mL CBD in Figure 6A. The orange “X” in Figure 6A indicates an example of a growth curve for a population that is developing too asynchronously to estimate an activity peak.

As originally designed, the wDAT evaluates the effects of continuous exposure from the onset of feeding after hatching through to the adult stage. To evaluate the reversibility of developmental delays and motility changes caused by CBD, an “early-only” exposure paradigm was also used. For the early-only exposures, approximately 3,000 worms in single wells were exposed for 24.5 ± 0.5 h from initial L1 feeding, followed by washing, re-feeding, and transfer to three wells for further monitoring (Figure 6B). Representative individual continuous vs early-only exposure wDAT experiments demonstrate that continuous exposure to purified CBD caused both hypoactivity and developmental delay (Figure 6A), while early-only exposures were associated with later developmental delays but not later hypoactivity (Figure 6B). Initial range-finding experiments found small and inconsistent effects at CBD concentrations of 300 μg/mL and below; therefore, the subsequent experiments used 300 μg/mL as the minimum developmental exposure.

In a set of four independent continuous exposure experiments, purified CBD induced delays of ∼5%–40% in a dose-response manner from 400 μg/mL to 1,000 μg/mL (Figure 6C). CBD-induced delays were relatively consistent across all four larval stages for most exposures, with the exception that the 5% delay to mid-L1 reached statistical significance at 300 μg/mL CBD, and delays to L2 tended to be both greater than delays to the other stages and more variable (Figure 6C). Higher concentrations of purified CBD induced some but not total, loss of synchronous developmental timing within the population; at 1,000 μg/mL CBD, an L4 peak had to be estimated in three out of four total experiments (Figure 6C, indicated by an orange “!3”). The effects on locomotion were quite different for different stages, with significant hypoactivity at the L1 and L4 stages with 400 μg/mL to 1,000 μg/mL purified CBD, L3 hypoactivity only at the highest concentration, and hyperactivity at L2 with 500 μg/mL and 750 μg/mL CBD (Figure 6D). The raw data for this set of experiments is provided in Supplementary Data Sheet 1.

**FIGURE 7:**
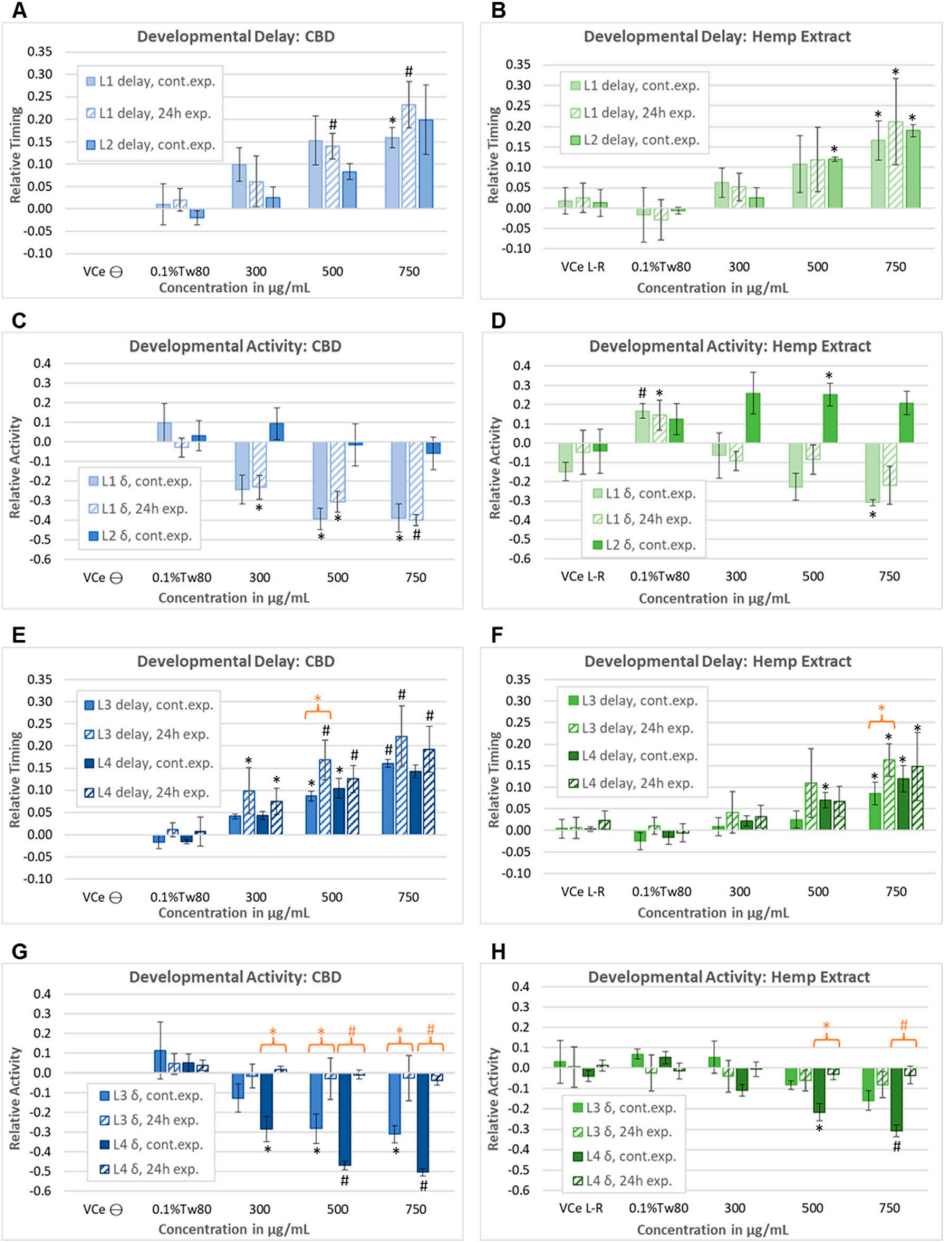
Assessment of the reversibility of adverse developmental outcomes. Effects of purified CBD and hemp extract at each of the four larval stages from continuous and early-only exposures are shown as solid and striped bars, respectively. Note that due to the timing of the washing steps in the early-only exposures, L2 data were evaluated for continuous exposures only. Effects of **(A)** purified CBD and **(B)** hemp extract on the time to reach mid-L1 and mid-L2. Effects of **(C)** purified CBD and **(D)** hemp extract on spontaneous locomotor activity at L1 and L2. **(E–H)** Effects of CBD and hemp extract on L3 and L4 developmental timing and activity. An orange asterisk or “#” symbol indicates where there was a significant difference between the continuous and early-only exposures at the same concentration. This figure represents five independently derived cohorts exposed to three sets of independently prepared emulsions for the early-only data. However, controls from two of the five continuous exposure experiments graphed in E–L did not meet acceptance criteria, so data were based on only three experiments for this set, which was reflected in higher *p*-values and lower confidence. For each experiment, the mean values across three replicate wells were normalized to the plate-matched vehicle emulsion (VCe) controls. VCe ϴ indicates control change values in the left plate, which are defined as zero, and VCe L-R indicates the difference between control wells in the left and right plates, giving an internal measure of experimental variability. Changes were considered meaningful if the mean difference from the plate-matched vehicle emulsion (VCe) control was ≥5% for delay and ≥10% for activity. Significant differences are indicated as Student’s t-test *p*-values of <0.05 (*) or <0.005 (#).

In a second set of wDAT experiments, continuous and early-only exposures for CBD and hemp extract were conducted simultaneously in order to distinguish reversible from irreversible developmental effects. For controls at 20°C in CeHM, the L1 stage lasts approximately 19 h and is entirely within the 24 h exposure period. Therefore, as expected, the L1 data from the continuous and early-only exposure data sets were not statistically different from each other (Figure 7A). For hemp extract exposures normalized to CBD concentration within the extract, L1, and L2 delays were similar to those seen with purified CBD (Figure 7B). As shown in Figure 6B, L2 locomotor activity was disrupted by washing and re-feeding; therefore, for L2, only the continuous exposure effects were evaluated. In this second set of experiments, purified CBD was not associated with significant L2 hyperactivity, but hemp extract was associated with hyperactivity at L2 (Figures 7C,D). The raw data for this set of experiments is provided in Supplemental Data Sheet 2.

At 20°C, control L3 activity peaks usually occur 38–40 h post initial L1 feeding, more than 12 h after removing the test article for early-only exposures. Not only were later delays not reduced by CBD removal, but there was a trend toward increased delays to L3 for early-only exposure populations (Figures 7E,F, striped bars) relative to continuous exposures (Figures 7E,F, solid bars). This trend achieved statistical significance at 500 μg/mL purified CBD, with L3 delays of 9% for continuous exposure but 17% with early-only exposure (Figure 7E, orange asterisk) and at 750 μg/mL CBD in hemp extract (Figure 7F, orange asterisk). In contrast to these irreversible effects on developmental delay, early-only CBD or hemp extract exposure was not associated with the later hypoactivity at L3 and L4 (Figures 7G,H), indicating that hypoactivity is a reversible effect.

In juveniles, the effects of the vehicle control emulsion relative to the oil-free control were inconsistent, with increased levels of activity with 0.1%Tw80 relative to VCe in some sets of experiments (Figures 6D, 7D) but not in others (Figures 7B,F,H).

### 3.6 CBD tissue concentrations

**FIGURE 8:**
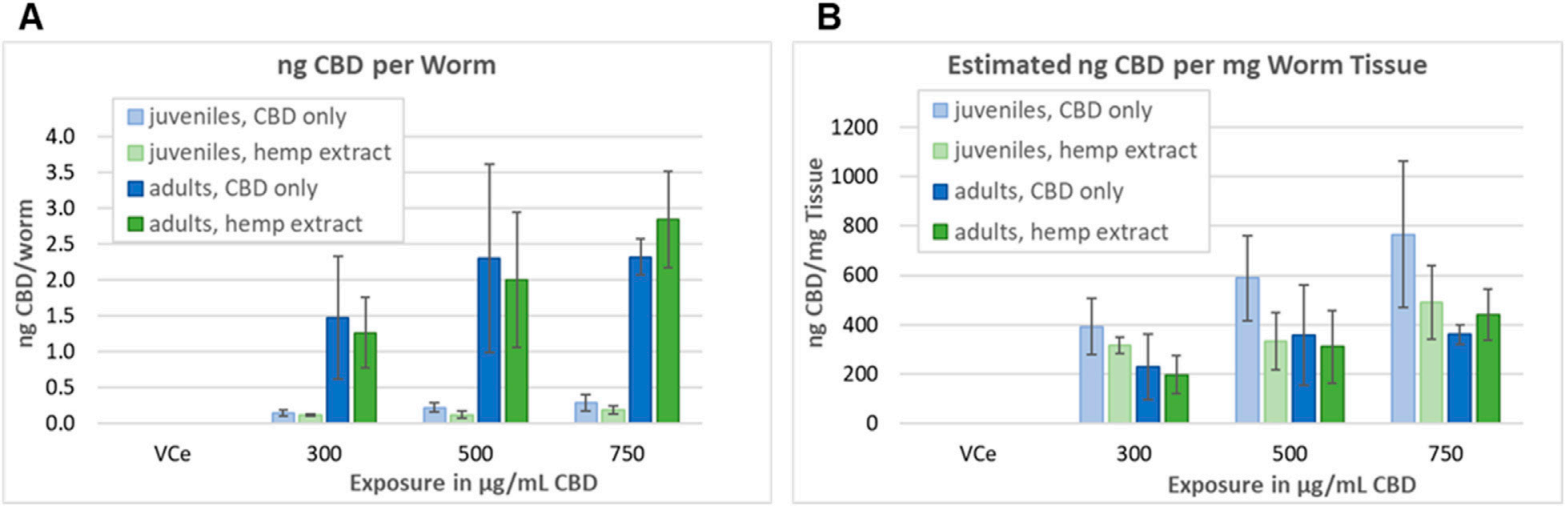
Assessment of tissue concentrations of CBD in *C. elegans*. **(A)** CBD concentrations in *C. elegans* exposed and analyzed as described in Section 2.8. Bars indicate the mean value of four independent experiments, and error bars indicate the standard deviations. **(B)** Concentration in ng CBD per mg *C. elegans* tissue, calculated as described in Section 3.6.

Adult and L1 *C. elegans* were exposed to CBD or hemp extract in emulsions for 24 h, then washed, homogenized, and assessed as described in Section 2.8 in four independent experiments. Mean internal CBD concentrations were 0.15, 0.22, and 0.29 ng CBD per juvenile *C. elegans* exposed to 300, 500, and 750 μg/mL purified CBD, respectively (Figure 8A). Although not statistically different from the purified CBD data set, the values were lower for hemp extract with 0.12, 0.13, and 0.19 ng CBD per juvenile worm exposed to hemp extract containing 300, 500, and 750 μg/mL CBD, respectively. Mean internal CBD concentrations were 1.47, 2.30, and 2.32 ng CBD per adult *C. elegans* exposed to 300, 500, and 750 μg/mL CBD, respectively (Figure 8A). For hemp extract, the values were 1.27, 2.00, and 2.85 ng CBD per adult worm for adults exposed for 24 h to hemp extract containing 300, 500, and 750 μg/mL CBD, respectively. Although the mean values indicate a trend toward increasing internal CBD concentrations with increasing exposure concentrations, only the adult worms exposed to 300 vs. 750 μg/mL CBD in hemp extract were statistically different from each other with a Student’s t-test *p*-value of <0.05. CBD was not detected in worms exposed to the vehicle control.

The volume of a juvenile *C. elegans* 24 h after L1 feeding (24hpL1f) has been calculated to be approximately 0.35 nL, and the volume of an adult to be approximately 6 nL (Knight et al., 2002). Therefore, a pellet containing approximately 10,000 adult *C. elegans* should have a volume of approximately 60µL, which is consistent with our observations. The density of well-fed L1 through L3 *C. elegans* has been measured to be approximately 1.091 g/mL and adult density to be approximately 1.074 g/mL 24 h after the L4 adult molt (Reina et al., 2013). Taking these published values together, the approximate mass/worm would be ∼0.38 µg for 24hpL1f *C. elegans* and ∼6.44 µg for adults. Dividing the nanogram CBD-per-worm values above by the calculated mass per worm, tissue concentrations ranged between 200–and 770 ng CBD per mg *C. elegans* tissue, with the highest tissue concentrations at each exposure being in juveniles exposed to purified CBD (Figure 8B). Although there was a trend toward higher CBD levels in purified CBD-exposed juvenile tissues, the juvenile *versus* adult and purified CBD *versus* hemp extract-exposed tissues were not statistically different at any tested concentration (Figure 8B).

## 4 Discussion

Edible products containing cannabidiol (CBD) or hemp extracts are widely available, and sales are increasing in the United States (Astray et al., 2023; Li et al., 2021; Saleska et al., 2022). Despite the widely perceived safety of CBD, its potential reproductive, developmental, and chronic exposure effects have not been sufficiently studied to determine safe levels for consumer use in foods and supplements (Argueta et al., 2020; Lee and Hardy, 2021; Henderson et al., 2023). Additionally, the effects of exposure to the mixtures of multiple cannabinoids in hemp extracts have not been adequately assessed for toxicologic interactions (Gingrich et al., 2023). The packaging of adult CBD doses into small edible products that resemble cookies or candy has increased the risk of accidental overdose in children and increased the need for more information on potential developmental and reproductive effects (Wong and Baum, 2019; Barrus et al., 2016).

Complex biological processes such as organismal developmental timing and chemically induced neurobehavioral effects cannot be fully modeled with currently available *in vitro* and microphysiological systems (Sachana et al., 2019; Blum et al., 2023). In contrast, concordance has been demonstrated for chemical effects in mammals and the microscopic nematode *Caenorhabditis elegans* for apical endpoints such as developmental delay and locomotor activity changes, in addition to chemical modes of toxic action (Masjosthusmann et al., 2018; Boyd et al., 2016; Cole et al., 2004; Hartman et al., 2021). Given that there is a broad conservation of endocannabinoid signaling across the animal kingdom (Pastuhov et al., 2016; Levichev et al., 2023; Paulsen and Burrell, 2019; Oakes et al., 2019), the *C. elegans* model may be useful for filling data gaps on the effects of cannabinoids and their mixtures.

Compared to testing with laboratory mammals, *C. elegans* can provide more rapid and less costly apical effect toxicity information (Hunt et al., 2020). One of the strengths of the *C. elegans* model for food-related chemical assessment is that it is primarily an oral toxicity model. In this study, emulsions were used to expose *C. elegans* to lipophilic CBD or hemp extract in an aqueous nutrient medium. Analysis of the prepared emulsions indicated that they had particles with similar mean negative charge and diameter compared to the oil-free control (Figure 2). This suggests that *C. elegans* responded to the emulsion particles in a similar manner to their normal *C. elegans* habitation medium food source.

High-fat diets (HFDs) are associated with increased oxidative stress in humans and other mammals (Le Lay et al., 2014; Matsuzawa-Nagata et al., 2008; Ding et al., 2023; Noeman et al., 2011). CBD reduces oxidative stress in human cell cultures, mice, rats, and *C. elegans*, at least in part through nuclear erythroid 2-related factor (Nrf2)-mediated transcriptional activation (Bielawiec et al., 2021; Zhang et al., 2022; Atalay et al., 2019; Juknat et al., 2013; da Cruz Guedes et al., 2023). The *C. elegans glutathione S-transferase 4* gene is often used as a bioreporter for the activation of oxidative stress response (OxStrR) gene expression, which is controlled by the *C. elegans* ortholog of Nrf2 (Blackwell et al., 2015). The vehicle control emulsion (VCe) carrier exposed the *C. elegans* to 0.5% sesame oil, a higher fat diet for this model (Gottschling and Doring, 2019). Compared to the oil-free control, VCe increased OxStrR bioreporter transgene expression approximately three-fold, and CBD at concentrations of 25 μg/mL and above reduced OxStrR in a dose-dependent manner (Figure 3A). At matched CBD concentrations of 25–500 μg/mL, hemp extract reduced OxStrR slightly more than purified CBD (Figure 3B). Additionally, there were small but statistically significant reductions in adult body size in this 24-h adult exposure assay for purified CBD at 400 μg/mL and above, while there were significant reductions in body size only at the highest tested concentration of 960 μg/mL CBD in hemp extract (Figure 3). Together, these data suggest that, as observed in humans and other mammals, HFD induces oxidative stress in *C. elegans* and that CBD in conjunction with other cannabinoids in hemp extract may be both more effective in reducing HFD-induced OxStrR and less toxic than purified CBD.

In mice, there is a clear association between HFD and hypolocomotion (Sharma and Fulton, 2013; Han et al., 2020; Wong et al., 2015; Branecky et al., 2015; Bjursell et al., 2008; Lavin et al., 2011). Some rat studies reported a correlation between HFD and reduced spontaneous locomotor activity, although others did not (Arika et al., 2019; Chia-Ying et al., 2018; Maric et al., 2022). In humans, correlations have been reported between higher fat intake and reduced physical activity, but it is not known whether the relationship is causal (Wong et al., 2015). In this study, exposure to the 0.5% sesame oil emulsion significantly reduced spontaneous locomotor activity in adult *C. elegans* relative to the oil-free control (Figure 4A). In juveniles, the HFD hypoactivity effect was not consistent, with slightly higher spontaneous locomotor activity in the oil-free-control-exposed cohorts relative to VCe in some experiments but not in others (Figures 6, 7).

Some studies have reported that CBD induces mild hyperlocomotion in rats, while others have found no CBD effects on behavior (Britch et al., 2017; Hlozek et al., 2017). CBD can ameliorate induced hypolocomotion in rodent models of arthritic inflammation, chronic liver disease, and Parkinson’s disease (Britch and Craft, 2023; Peres et al., 2016; Magen et al., 2009). Previous studies in *C. elegans* have demonstrated that both CBD and its related compound cannabigerol can improve healthspan by reducing the hypoactivity effect of aging in chronically exposed older cohorts (Kulpa et al., 2023; Land et al., 2021). Additionally, CBD has been reported to improve motor impairment and behavioral alterations in *C. elegans* models of Alzheimer’s and Parkinson’s disease (Zhang et al., 2022; Wang et al., 2021; Muhammad et al., 2022; da Cruz Guedes et al., 2023). In this study, purified CBD at 200–500 μg/mL restored locomotor activity in emulsion-exposed adult *C. elegans* to oil-free control levels within 8–12 h (Figure 4A). In contrast, locomotor activity remained at oil-free control levels for all exposures of CBD in hemp extract throughout the 18-h exposure and assessment period (Figure 4B), suggesting that CBD-rich hemp extracts may be more effective than purified CBD in ameliorating HFD-induced hypoactivity in adult *C. elegans*.

Endocannabinoids play a variety of roles in male and female reproductive systems (Tirado Munoz et al., 2020). CBD has been associated with adverse reproductive effects in mammals, primarily in males and at exposures higher than those recommended for humans (Huestis et al., 2019; Carvalho et al., 2020). Reproduction in *C. elegans* is not directly comparable to mammalian reproduction. For example, gonadotropin hormones are not conserved in invertebrates, *C. elegans* do not have hormone-regulated cyclical fertility periods, nor do they form a placenta around embryos, and *C. elegans* sperm have a pseudopod instead of a flagellum (Athar and Templeman, 2022; L'Hernault, 2006). However, many aspects of meiosis and oocyte maturation and quality control are conserved from *C. elegans* to mammals, including the participation of conserved cell-cycle regulators, intracellular organelle transfer through a shared cytoplasm, the use of conserved apoptotic pathways to remove damaged oocytes, the importance of mitochondrial function in maintaining oocyte quality, the requirement for efficient telomerase activity, the increased incidence of chromosomal non-disjunction with aging, and the role of insulin-like growth factor 1 signaling and FOXO3/DAF-16 activity in maintaining oocyte quality and conserving fertility (Athar and Templeman, 2022; Greenstein, 2005; Das and Arur, 2017). Additionally, many processes involved in embryogenesis are also conserved from nematodes to humans, including genes for key signaling cascades involved in organismal development, the cellular and molecular mechanisms underlying embryonic cell patterning, and histone modification for the transfer of epigenetic information (Arico et al., 2011; Leung et al., 2008). Therefore, while reduced reproductive output in *C. elegans* cannot directly predict human reproductive-specific effects, it can model more generalized forms of conserved toxicity mechanisms that interfere with oocyte production, embryonic development, and progeny survival.

To assess CBD and hemp extract chronic exposure effects on reproduction and body size in gravid *C. elegans* adults and their progeny, synchronized populations were evaluated on days 2, 3, and 4 of continuous exposure from the onset of reproductive maturity. The reproductive period in *C. elegans* hermaphrodites lasts for approximately 6 days (Johnson, 1987), so these data represent progeny output for just over half of the reproductive span of the model. Purified CBD significantly reduced the ratio of progeny to adults in a dose-dependent manner at 400 μg/mL and above (Figure 5A). Compared to plate-matched VCe controls, the ratio of progeny to adults for each concentration of purified CBD was relatively consistent over the 3 days of assessment. In contrast, CBD in hemp extract reduced progeny output only at 750 μg/mL and above, and only on days 2 and 3 of exposure (Figure 5B). By day 4 of parental exposure, the ratio of progeny to adults at all tested CBD concentrations in hemp extract was not significantly different from the matched emulsion or oil-free controls. Together, these data are consistent with reduced reproductive toxicity for the hemp extract relative to purified CBD and suggest a possible adaptation to the hemp extract over time for reproductive output.

Purified CBD significantly reduced *C. elegans* parental body size by approximately 10%–30% in a dose-dependent manner at 500 μg/mL and above (Figure 5C). Smaller reductions of approximately 5% in parental body size were also seen at 300 and 400 μg/mL purified CBD, but these were significant only on day 3 of exposure. With continuous exposure to purified CBD, adult-size effects were relatively consistent across the 3 days of assessment. In contrast, for CBD in hemp extract, smaller parental body size reductions of 5%–14% were seen at 500 μg/mL CBD and above, and these were statistically significant only on day 2 of exposure (Figure 5D)—again suggesting potential adaptation to hemp extract exposure in adults. In stark contrast to adults, longer population exposure resulted in further reductions in mean juvenile body size. For purified CBD, significant reductions in mean progeny size were seen at 500 μg/mL on day 2 of parental exposure, 400 μg/mL on day 3, and 200 μg/mL on day 4 (Figure 5E). CBD in hemp extract had smaller effects on progeny size and at higher concentrations than purified CBD, but as with purified CBD, mean progeny size further decreased with longer exposure to hemp extract (Figure 5D). Because progeny output recovered somewhat by day 4 of population exposure to CBD in hemp extract (Figure 5B), further reduced mean progeny body size over time could be explained by a delay in parental egg laying. However, because there was little change in progeny output at each concentration of purified CBD over time (Figure 5A), the reductions in mean progeny size seen with purified CBD (Figure 5E) are more likely to have been caused by CBD-related developmental toxicity. This cumulative effect on the mean progeny population body size could result from chronic parental exposure during the oogenesis and embryogenesis of later progeny, although further research would be needed to verify this hypothesis.

Maternal cannabis use during pregnancy in humans is associated with low birth weight (Gunn et al., 2016). In studies in mice, rabbits, and rats, high maternal CBD exposure reduced maternal body weight in addition to fetal and birth weight and/or neonatal growth (Huestis et al., 2019; Fish et al., 2019; Henderson et al., 2023; Gingrich et al., 2023; Tallon and Child, 2023). Multiple aspects of endocannabinoid signaling, including the regulation of organismal development, neuronal development, and the regulation of specific behaviors, are well conserved from worms to humans (Pastuhov et al., 2016; Estrada-Valencia et al., 2023). Cohorts of *C. elegans* continuously exposed to purified CBD from first feeding to adulthood had reduced growth rates in dose-response from 300 μg/mL to 1,000 μg/mL CBD (Figures 6, 7). The effects of continuous exposure to CBD effects on juvenile spontaneous locomotor activity appeared to be stage-dependent, with significant hypoactivity in the first, third, and fourth larval stages, but hyperactivity in some experiments at the second larval stage for both purified CBD and hemp extract (Figures 6, 7). As with other assays in this study, CBD in hemp extract was less toxic than purified CBD, with a LOAEL for developmental toxicity of 500 μg/mL (Figure 7). With early-only CBD exposures, later juvenile locomotor activity did not significantly differ from oil-free and vehicle emulsion controls (Figures 6B, 7G), consistent with the reversibility of locomotor effects. In striking contrast, delays in timing to reach later developmental stages were not reversed with early-only CBD exposure (Figures 6B, 7E), consistent with irreversible effects on developmental timing. Not only were the developmental delays induced by CBD irreversible but there was a trend to greater delays to the third larval stage (L3) after early-only exposures relative to continuous exposure (Figures 7E,F). This finding may indicate that there is a time lag in the adaptation of essential endocannabinoid signaling after an exogenous cannabinoid has been removed; however, an assessment of endocannabinoid levels would be needed to verify this hypothesis.

Internal CBD concentrations in 24 h exposed *C. elegans* juveniles and adults were measured, and ng CBD per worm and ng/mg tissue were estimated, as described in Sections 2.8 and 3.6. *C. elegans* juveniles exposed to purified CBD had the highest calculated CBD tissue concentrations at 400–770 ng/mg (Figure 8). Adults and juveniles exposed to hemp extract had lower tissue concentrations of 200–490 ng/mg, but these values are still two- to four times higher than those seen in adipose tissue in a 28-day study of oral CBD exposure in rats at human-relevant doses (Child and Tallon, 2022), and higher by two orders of magnitude or more than those seen in other tissues in mammals (Gingrich et al., 2023). These findings suggest that the toxicities seen in this study are caused by exposures that far exceed recommended human dose levels.

In this study, compared to purified CBD, hemp extract at matched CBD concentrations had a) reduced effects on adult and juvenile body size, b) higher LOAELs for developmental endpoints, and c) slightly greater reductions in HFD-induced oxidative stress. These findings in *C. elegans* are consistent with a meta-analysis of published studies in patients with seizures using either purified CBD or CBD-rich extracts (Pamplona et al., 2018). Pamplona and colleagues found that there were a) fewer reports of adverse effects from patients, b) more reports of reduced seizure frequency, and c) similar improvements at lower doses in patients treated with CBD-rich extracts relative to those treated with purified CBD. Although the human endpoints from this meta-analysis are not directly comparable to the effects in *C. elegans*, both studies indicate that CBD-rich hemp extracts may be less toxic than purified CBD.

The use of non-mammalian *in vivo* models is encouraged as supporting data in the weight of evidence evaluations, and evidence of the irreversibility of developmental effects is a cause for increased concern in developmental and reproductive risk assessment (EMA/CHMP/ICH, 2020). In this study, CBD, alone or in hemp extract, was more toxic to juveniles than it was to adults, reductions in body size in juveniles were cumulative with chronic population exposures beginning at parental reproductive maturity, and early developmental exposure caused irreversible delays in later developmental stages. For each tested *C. elegans* endpoint, a CBD-rich hemp extract was at least slightly less toxic than purified CBD. Dosimetry indicated that the *C. elegans* LOAELs for all toxicity endpoints were significantly higher than the recommended CBD patient dosing levels.

## Data Availability

The original contributions presented in the study are included in the article/Supplementary Material; further inquiries can be directed to the corresponding author.

## Abbreviations

1%Tw80: 1% Tween (polysorbate) 80 in non-fat cow’s milk; 0.1%Tw80: a 1:9 mix of 1%Tw80 in CeHM; 2-AG: 2-arachidonoylglycerol; AEA: anandamide; CBD: cannabidiol; CeHM: *C. elegans* habitation medium; a 1:4 mix of non-fat cow’s milk and chemically defined nutrient medium; HFD: high-fat diet; LOAEL: lowest observed adverse effect level; NOAEL: no observed adverse effect level; OxStrR: transcriptional oxidative stress response; TOF: time-of-flight, a measure of body size; VCe: vehicle control emulsion; wAAT: worm Adult Activity Test; wDAT: worm Development and Activity Test.
