# Supplementary material for: Assessment of the effects of cannabidiol and a CBD-rich hemp extract in Caenorhabditis elegans
Source: Front Toxicol. 2024 Oct 1;6:1469341. doi: 10.3389/ftox.2024.1469341 (PMC11484448; doi:10.3389/ftox.2024.1469341)
Supplement: Supplementary file 1 [file Table1.docx]

**Hermaphrodite (female program) ReproTox Assay: CBD**

| Test Articles | Abbrev. | MW | CAS # | Notes |
| --- | --- | --- | --- | --- |
|  |  |  |  | ‎ |
| Cannabidiol | CBD | 314.5 | 13956-29-1 |  |

| **strain: PD4251 thaw date: CeHR prep date: Milk:** | | | | | | | | |
| --- | --- | --- | --- | --- | --- | --- | --- | --- |
|  | **hpL1f** | **Day** | **Day** | **hrs.** | **Date** | **Time** | **Notes** | **Who** |
| Feed |  |  |  |  |  |  |  |  |
| Egg Prep | -20 | -1 | Thr. | -20 |  | noon |  |  |
| Feed EP | 0 | 0 | Fri. | 0 |  | 8am |  |  |
| Monitor | 24 | 1 | Sat. | 24 |  |  |  |  |
| Monitor | 48 | 2 | Sun. | 48 |  |  |  |  |
| Dose | ~82 | 3 | Mon. |  |  | 4-8pm |  |  |
| Monitor | 96 | 4 | Tue. |  |  |  |  |  |
| COPAS | 120 | 5 | Wed. |  |  |  |  |  |
| COPAS | 144 | 6 | Thr. |  |  |  |  |  |
| COPAS | 168 | 7 | Fri. |  |  |  |  |  |

**Prep Day**

- Feed healthy PD4251 cultures with CeHM, maintain at **19°C** (allows for all steps to occur between 8am and 8pm)

**Egg Prep, Afternoon Day -1**

- Put CeHR, milk, 0.1N NaCl, bleach, 5N NaOH, and M9 in hood
- Turn on centrifuge and set to 1,000 x g and 1min
- Wipe down hood, centrifuge, frig and incubator handles with 70% alcohol
- Update *C. elegans* maintenance table
- Time from 1^st^ wash in 0.1N NaCl to bleach addition should be 20min max
- Worms in 0.1N NaCl *start time/hood temp* ________________
- Settle and wash PD4251 gravid adults with 0.1N NaCl 3x □ □ □
- Bleach brand:__________, bleach open date__________, add bleach  *time*__________
- 1^st^ spin in hypochlorite  *time*­­­­__________, 1^st^ spin in M9  *time*__________
- M9 washes □ □ (1 is enough if pellets are nearly dry)
- Egg Prep finished and in M9 *time* _________ + 20h (@19°C) =
- Egg quality report:

| **Prep:**  **Dauers:**  **Quality:**  **Count:** | **Prep:**  **Dauers:**  **Quality:**  **Count:** |
| --- | --- |

**Feed L1s, Morning Day 0**

- Put water, Parmalat, and CeHR in the hood to warm up
- Wipe down hood, centrifuge, benches, handles with 70% alcohol
- Assess Egg Prep and update *C. elegans* maintenance table
- Set centrifuge to 3,000 x g for 5 min at 18°C
- L1 Quality, % unhatched eggs, dauers __________________________
- Centrifuge and then check L1 pellet in scope, spin again if moving L1s are above pellet
- Aspirate M9 supernatant from L1s
- Resuspend worms to <1,000 worms/mL CeHM  *time*__________
- Return to 19°C incubator

Count: 1. _____________________________________________ = _____ worms/mL

Count: 2. _____________________________________________ = _____ worms/mL

Count: 3. _____________________________________________ = _____ worms/mL

**Monitor Developmental Timing in Flask(s) and After Plating (p3)**

- Dose when at least 50% of cohort have a visible primary oocyte, and <20% have L4 gonadal morphology. See (Seydoux et al. 1993) for detailed L4 gonadal structure identification. At 19°C, this should occur at about 82h post L1 feeding (82hpL1f), but monitoring should be done every time.

**Plate Prep for Monitoring and Dosing**

- Put water, Parmalat, and CeHR in the hood
- Wipe down hood, centrifuge, benches, handles with 70% alcohol
- Update *C. elegans* maintenance table
- Remove PD4251s from flasks and allow to settle
- Refeed and count, dilute to ~250 yAs/mL CeHM

Count: 1. _____________________________________________ = _____ worms/mL

Count: 2. _____________________________________________ = _____ worms/mL

Count: 3. _____________________________________________ = _____ worms/mL

- Load 900µL worms in CeHM-Parm into wells of 8 (2 extra) plates in designated pattern
- Do not dose until the majority of worms in wells have reached the young adult stage


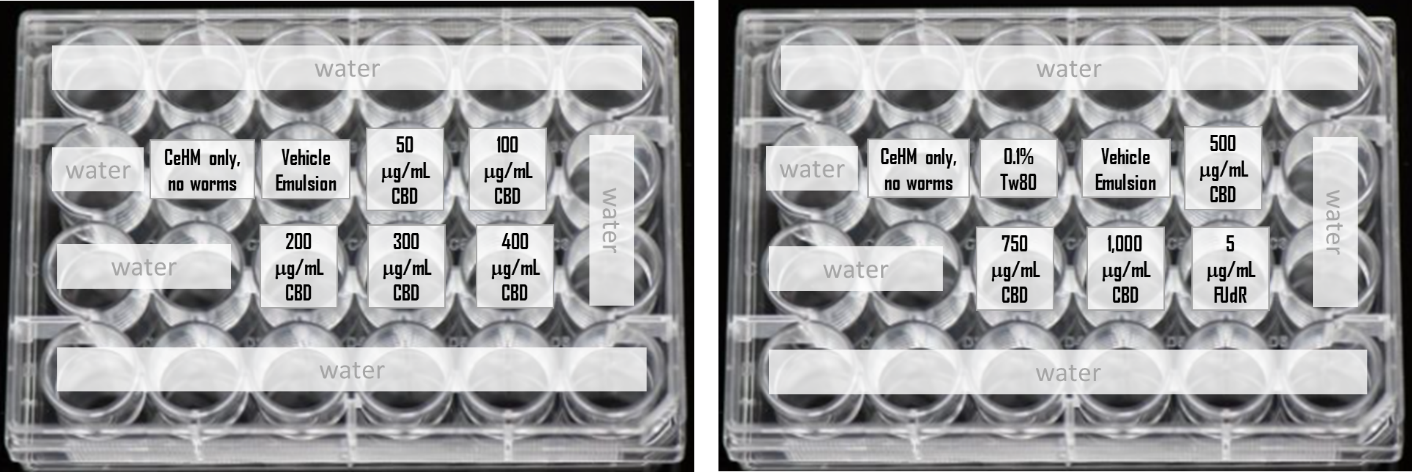


**Monitor Developmental Timing and Dose, Day 3pL1f**

- Add 100μL of 10x dosing solutions in the pattern indicated
- Agitate plates by hand to ensure mixing
- Only touch edges of plates, preferably the corners = avoid transferring body heat to wells
- Positive control: 1^st^ add 100µL Vehicle Control emulsion swirl, then add 5µL of 1mg/mL FUdR, swirl again
- Mark finish dosing time here and on p1 table *time* __________
- Return plates to 19°C incubator on shakers set to 120rpm

**Notes and Images, day 5 post L1 feeding (d5pL1f), day 2 post dosing (d2pD)**

- Examine wells in microscope, TAKE NOTES

| Well | Final | Notes |  |  |  |  |  |
| --- | --- | --- | --- | --- | --- | --- | --- |
| p1 B3 | vehicle control emulsion |  |  | **hpL1f** |  |  |  |
| p1 B4 | 50µg/mL |  | Fri. | ***time*** |  |  |  |
| p1 B5 | 100µg/mL |  | Sat. | 24 |  |  |  |
| p1 C3 | 200µg/mL |  | Sun. | 48 |  | **hpD** |  |
| p1 C4 | 300µg/mL |  | Mon. | 72 |  | ***time*** |  |
| p1 c5 | 400µg/mL |  | Tue. | 96 |  | 24 |  |
| p2 B3 | 0.1%Tw80 in Parm |  | Wed. | 120 |  | 48 |  |
| p2 B4 | vehicle control emuls.#2 |  | Thr. | 144 |  | 72 |  |
| p2 B5 | 500µg/mL |  | Fri. | 168 |  | 96 |  |
| p2 C3 | 750µg/mL |  |  |  |  |  |  |
| p2 C4 | 1mg/mL |  |  |  |  |  |  |
| p2 C5 | vehicle emuls. + 5µg/mL FUdR |  |  |  |  |  |  |

**COPAS, Day 5pL1f**

- Turn on 5 instruments: air compressor, laser box, reader/sorter, LPS, and computer
- Select LPS+Biosorter icon – will take a few minutes for software to start
- **Add 150µL M9** to all experiment wells
- Remove water and add COPAS cleaning solution as indicated in diagram
- Confirm sheath fluid (behind LPS) and wash bottles (water) are full, waste bottle empty
- In FlowPilotII window, set up graphs, set TOF min to 100, and select “Enable Lasers”
- In LP Sampler window, select PRIME to remove air from tubing
- Click PLATE TEMPL to select wells for analysis
- CBD Plate 1 Day 5, _______h post L1 feeding and _______h post dosing
-
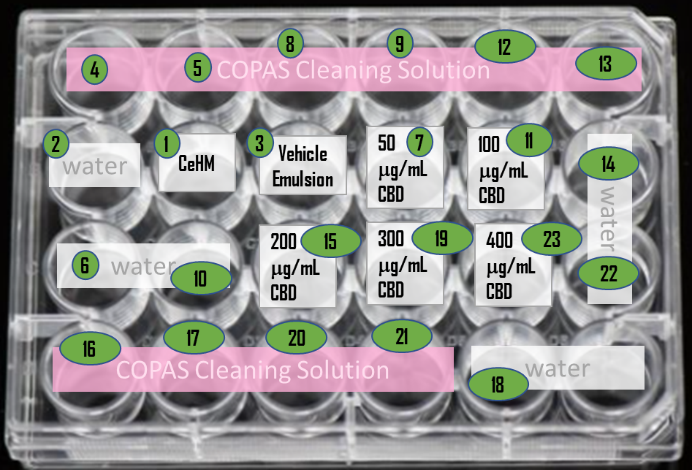
Run a plate with 6 wells each of COPAS cleaning solution and water to clean lines
- CBD Plate 2 Day 5, _______h post L1 feeding and _______h post dosing
- Data check in excel – use the extra plates if necessary
- When done, wash with bleach□, cleaning solution□, 70% isopropanol□, DI water□
- Turn off all 5 instruments, refill cleaning solutions

**Notes and Images, d6pL1f, d3pD**

- Examine wells in microscope, TAKE NOTES

**CBD Plates Day 6pL1f Notes**

| Well | Final | Notes |  |  |  |  |  |
| --- | --- | --- | --- | --- | --- | --- | --- |
| p1 B3 | vehicle control emulsion |  |  | **hpL1f** |  |  |  |
| p1 B4 | 50µg/mL |  | Fri. | ***time*** |  |  |  |
| p1 B5 | 100µg/mL |  | Sat. | 24 |  |  |  |
| p1 C3 | 200µg/mL |  | Sun. | 48 |  | **hpD** |  |
| p1 C4 | 300µg/mL |  | Mon. | 72 |  | ***time*** |  |
| p1 c5 | 400µg/mL |  | Tue. | 96 |  | 24 |  |
| p2 B3 | 0.1%Tw80 in Parm |  | Wed. | 120 |  | 48 |  |
| p2 B4 | vehicle control emuls.#2 |  | Thr. | 144 |  | 72 |  |
| p2 B5 | 500µg/mL |  | Fri. | 168 |  | 96 |  |
| p2 C3 | 750µg/mL |  |  |  |  |  |  |
| p2 C4 | 1mg/mL |  |  |  |  |  |  |
| p2 C5 | vehicle emuls. + 5µg/mL FUdR |  |  |  |  |  |  |

**COPAS, Day 6pL1f**

- Turn on 5 instruments: air compressor, laser box, reader/sorter, LPS, and computer
- Select LPS+Biosorter icon – will take a few minutes for software to start
- **Add 200µL M9** to all experiment wells
- Remove water and add COPAS cleaning solution as indicated in diagram
- Confirm sheath fluid (behind LPS) and wash bottles (water) are full, waste bottle empty
- In FlowPilotII window, set up graphs, set TOF min to 100, and select “Enable Lasers”
- In LP Sampler window, select PRIME to remove air from tubing
- Click PLATE TEMPL to select wells for analysis
- Once plate is in place and **lid removed**, click RUN PLATE, select folder and name file
- Extract Plate 1 Day 6, _______h post L1 feeding and _______h post dosing
- Run a plate with 6 wells each of COPAS cleaning solution and water to clean the lines
- Extract Plate 2 Day 6, _______h post L1 feeding and _______h post dosing
- Data check in excel – use the extra plates if necessary
- When done, wash with bleach□, cleaning solution□, 70% isopropanol□, DI water□
- Turn off all 5 instruments, refill cleaning solutions

**Notes and Images, d7pL1f, d4pD**

- Examine wells in microscope, TAKE NOTES

**CBD Plates Day 7pL1f Notes**

| Well | Final | Notes |  |  |  |  |  |
| --- | --- | --- | --- | --- | --- | --- | --- |
| p1 B3 | vehicle control emulsion |  |  | **hpL1f** |  |  |  |
| p1 B4 | 50µg/mL |  | Fri. | ***time*** |  |  |  |
| p1 B5 | 100µg/mL |  | Sat. | 24 |  |  |  |
| p1 C3 | 200µg/mL |  | Sun. | 48 |  | **hpD** |  |
| p1 C4 | 300µg/mL |  | Mon. | 72 |  | ***time*** |  |
| p1 c5 | 400µg/mL |  | Tue. | 96 |  | 24 |  |
| p2 B3 | 0.1%Tw80 in Parm |  | Wed. | 120 |  | 48 |  |
| p2 B4 | vehicle control emuls.#2 |  | Thr. | 144 |  | 72 |  |
| p2 B5 | 500µg/mL |  | Fri. | 168 |  | 96 |  |
| p2 C3 | 750µg/mL |  |  |  |  |  |  |
| p2 C4 | 1mg/mL |  |  |  |  |  |  |
| p2 C5 | vehicle emuls. + 5µg/mL FUdR |  |  |  |  |  |  |

**COPAS, Day 7pL1f**

- Turn on 5 instruments: air compressor, laser box, reader/sorter, LPS, and computer
- Select LPS+Biosorter icon – will take a few minutes for software to start
- **Add 250µL M9** to all experiment wells
- Remove water and add COPAS cleaning solution as indicated in diagram
- Confirm sheath fluid (behind LPS) and wash bottles (water) are full, waste bottle empty
- In FlowPilotII window, set up graphs, set TOF min to 100, and select “Enable Lasers”
- In LP Sampler window, select PRIME to remove air from tubing
- Click PLATE TEMPL to select wells for analysis
- Once plate is in place and **lid removed**, click RUN PLATE, select folder and name file
- CBD Plate 1 Day 7, _______h post L1 feeding and _______h post dosing
- Run a plate with 6 wells each of COPAS cleaning solution and water to clean the lines
- CBD Plate 2 Day 7, _______h post L1 feeding and _______h post dosing
- Data check in excel – use the extra plates if necessary
- When done, wash with bleach□, cleaning solution□, 70% isopropanol□, DI water□
- Turn off all 5 instruments, refill cleaning solutions

**Reference**

- Seydoux, G., Savage, C., and Greenwald, I. (1993). Isolation and characterization of mutations causing abnormal eversion of the vulva in *Caenorhabditis elegans*. Dev Biol. 157, 423-36. <https://doi.org/10.1006/dbio.1993.1146>.
