# Supplementary material for: Assessment of the effects of cannabidiol and a CBD-rich hemp extract in Caenorhabditis elegans
Source: Front Toxicol. 2024 Oct 1;6:1469341. doi: 10.3389/ftox.2024.1469341 (PMC11484448; doi:10.3389/ftox.2024.1469341)
Supplement: Supplementary file 2 [file Table2.docx]

**Emulsion Prep Date:**

**Calculate Volume of Each Dosing Solution Needed:**

- 1,200µL for dosing 12 wells for juvenile worms/concentration
- 1,200µL for dosing 12 wells for adult worms/concentration
- 600µL for dosing wDAT x2
- 200µL for dosing wDAT+ x2
- = 3.2mL needed for each test article

**The LV1 microfluidizer (MF) has a capacity of 6mL per batch.**

**Calculate Volume Vehicle Control Emulsion (VCe) Needed:**

- 100µL/well x 12 wells/plate x 6 plates = 7.2mL for juvenile standard curve
- 100µL/well x 12 wells/plate x 6 plates = 7.2mL for adult standard curve
- Plus 17.6mL for control dosing and dilutions ∴ 32mL total, so make 7 batches for 42mL VCe

**Calculate Number of L1 Worms Needed (x # of Test Articles):**

- wDAT and wDAT+: (1,000 L1s/well x 12 wells/plate x 2 plates) + (10 wells x 3,000 L1s/well) = 54k L1 worms
- Worm Concentrations: 12 wells/plate x 3,000 L1s/well x 4 plates x 2 (CBD+extr.) for dosing = 288k L1 worms
- Calibration Curve ‘Matrix’ Prep: 12 wells/plate x 3,000 L1s/well x 6 plates = 216k worms, ∴ **558k L1s total**

**Calculate Number of yA worms needed:**

- Worm Concentrations: 12 wells x 1,000 yAs/well x 4 plates x 2 Test Articles for dosing = 96k yA worms
- Calibration Curve ‘Matrix’ Prep: 12 wells x 1,000 L1s/well x 6 plates = 72k yA worms ∴ 168k yA total

**CBD**

| 10x Dosing Solutions | Vehicle Emulsion | 10mg/mL CBD | Dosing Solution volume made | Dosing Solution volume required | Final Exposure Concentration |
| --- | --- | --- | --- | --- | --- |
| 1%Tw80 in Parm | X | X |  | 800µL | 0.1%Tw80 |
| emulsion | 3.2mL | X |  | 3.2mL | 0.5% oil, 0.095%Tw80 |
| 3mg/mL | 2.8mL | 1.2mL | 4mL | 3.2mL | 300µg/mL CBD |
| 5mg/mL | 2.0mL | 2.0mL | 4mL | 3.2mL | 500µg/mL CBD |
| 7.5mg/mL | 1.0mL | 3.0mL | 4mL | 3.2mL | 750µg/mL CBD |
| Total Needed | **9mL =**  **2 batches** | **6.2mL** |  | **13.6mL** |  |

**Extract**

Extract has 175,415µg CBD/mL

175,415µg ÷ 20mL = 8.771mg CBDeq./mL

For 3.5mL of **7.5mg/mL** = 26.25mg | 26.25mg ÷ 8.771mg/mL = 2,993µL needed

For 3.5mL of **5mg/mL** = 17.5mg | 17.5mg ÷ 8.771mg/mL = 1,995µL needed

For 3.5mL of **3mg/mL** = 10.5mg | 10.5mg ÷ 8.771mg/mL = 1,197µL needed

| 10x Dosing Solutions | Vehicle Emulsion | Extract | Dosing Solution volume made | Dosing Solution volume required | Final Exposure Concentration |
| --- | --- | --- | --- | --- | --- |
| 1%Tw80 in Parm | X | X |  | 800µL | 0.1%Tw80 |
| emulsion | 3.2mL | X |  | 3.2mL | 0.5% oil, 0.095%Tw80 |
| 3mg/mL | 2,303µL | 1,197µL | 3.5mL | 3.2mL | 300µg/mL CBD |
| 5mg/mL | 1,505µL | 1,995µL | 3.5mL | 3.2mL | 500µg/mL CBD |
| 7.5mg/mL | 507µL | 2,993µL | 3.5mL | 3.2mL | 750µg/mL CBD |
| Total Needed | **8.6mL =**  **2 batches** | **6.2mL** |  | **13.6mL** |  |

- *Make 20% extra 1% tween 80 in Parmalat to account for foam*
- *Also make extra vehicle emulsion mix so we aren’t trying to get the last drops at the bottom of the conical tube into the syringe*

**The Day Before**

- Thaw Parmalat in refrigerator

**Amount 1% Tween 80 in Parmalat**

- 1%Tw80 Non-Fat Control:
  - 1.6mL of 1%Tw80 in Parmalat for control dosing
- VCe + microfluidizer washes:
  - 4x6mL batches of vehicle emulsion for toxicity testing
  - 3x6mL batches of vehicle emulsion to expose worms to create standard curves for chemistry
  - 5x6mL to clean the microfluidizer and toss = 72mL of 1%Tw80 for the sesame/milk mix needed
- Plus, another 40mL of 1%Tw80 for the two dosing emulsions
- Therefore, prep 140mL of 1% Tween 80 in milk

**Mixture Preps**

- Wear face mask and hair net in addition to lab coat and gloves
- Set heat block to 37°C and put a clean 200mL beaker ~1/3 full of autoclaved MilliQ water on top of it
- Set second heat block to 100°C and put a sterile Erlenmeyer flask of autoclaved MilliQ water on top of it
- Put 1mL tips, Parmalat, Tween 80, sesame oil, CBD, and hemp extract in hood
- Store sesame oil at 4°C and use within 2 months of opening bottle
- Set out microfluidizer cleaning solutions, then wipe down work areas with 70% alcohol including hood, frig and incubator handles, microfluidizer, countertops, and ice buckets
- 200mg CBD in 1mL Sesame Oil
  - Pour CBD powder from two 100mg vials into a 50mL conical
  - Tap vial gently inside conical, rotate and repeat
  - Then, vigorously tap vial into the cap of the 50mL conical to remove as much CBD as possible
  - Finally, remove the rest of the CBD with the 1mL sesame oil and add to the 50mL conical
  - Put the 200mg CBD in 1mL sesame oil in the 37°C water, and periodically vortex and centrifuge until dissolved – this can take up to an hour
- Add 200µL of Tween 80 to seven empty sterile 50mL conicals
- Prep 140mL of 1% Tween 80 in milk = 7 x (0.2mL Tw80 in 19.8mL milk)
- Agitate 1%Tw80 in Parm well, taking enough time to make sure that the Tween 80 is dissolved
- **Vehicle Control:** for 5% oil, mix 1mL sesame oil with 19mL 1%Tw80 in Parm 4x for 80mL mix
- **CBD:** add 19mL of 1%Tw80 to 1mL CBD in oil = 20mL of 5mg/mL CBD in 0.5% sesame oil/0.95%Tw80
- **Extract:** add 19mL of 1%Tw80 to 1mL extract = 20mL of 5mg/mL extract in 0.5% sesame oil/0.95%Tw80

**Using the LV1: Preparation**

- Prep hot autoclaved water, hot and RT cleaning solutions, 70% isopropanol
- Have enough ice on hand to pile around the microfluidizer core and more ice in ice bucket for samples
- Put LV1s ice holder drain hose into a collection beaker
- Spray everything you will touch, or the syringes will touch, with 70% isopropanol

**Using the LV1: Starting the Machine**

1. Main power switch is low, behind the left side as you face the machine – turn this on
2.
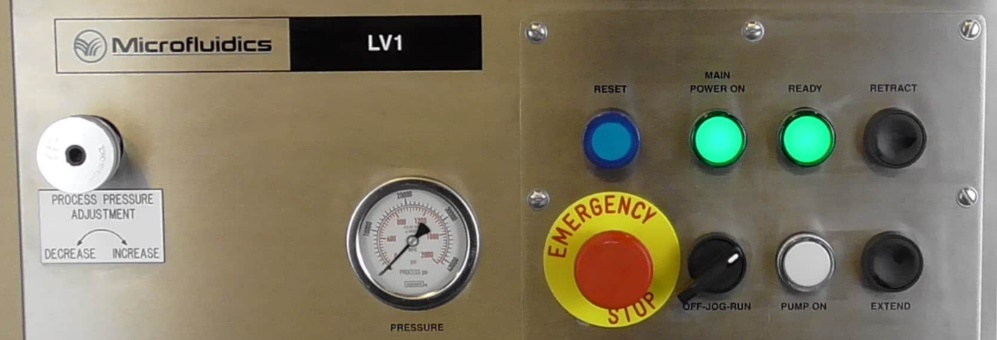
This will make the RESET and MAIN POWER ON buttons light up
3. Press the blue RESET button, which will make that light turn off
4. Turn the mode switch to JOG, which should turn the motor on
5. Press and hold the EXTEND button for ~15 seconds to fully extend the piston

**Running a sample**

1. To set the pressure level:
   1. turn the mode switch to RUN (as shown above)
   2. this will increase the pressure and the PUMP ON light will come on
   3. wait until the Pump On light goes off and the green READY light goes on
   4. hold in the black EXTEND button (bottom right = “process button”) while turning the silver pressure control nob (at left) until the gage reads ~10% above the desired pressure
   5. release the EXTEND “process button” and turn the mode switch to JOG
   6. wait for pressure release and the hissing noise to stop (the PRESSURE gage will go back to zero)
2. Attach empty collection syringe to right side of the interaction chamber
3. Draw more sample than needed into a Luer Lock syringe and attach to inlet connection
4. Press and hold the RETRACT button while (if necessary) depressing syringe
5. Release RETRACT button when desired volume is reached (max = 6mL)
6. Turn switch to RUN position
7. Wait for the PUMP ON light to go off and the READY light to come on
8. Push and hold the EXTEND button until the product has moved into the collection syringe
9. Return mode switch to JOG position
10. Manually check temperature of emulsion – if warm, put on ice before running through another cycle

**Cleaning Before Use**

- Follow step 1 above, setting to 20,000psi
- Follow steps 2-9 above 3x with water to remove isopropanol
- Run 3x with room temperature Zymit solution
- Run 3x with hot Dawn solution (blue)
- Run with hot autoclaved MilliQ water until solution runs clear, 8x minimum
- MACHINE NEEDS TO COOL DOWN AFTER 120 CYCLES (maximum use), ~20 cycles at this point

**Making Emulsions**

- Think about workflow and make sure everything you do will keep emulsions as sterile as possible
- Vortex mixture preps for at least 60 seconds prior to pulling into syringes
- Reset **PSI to 10k**, and run each dosing solution through the LV1 8x
- Vehicle Control Emulsion (VCe) batches can be run as doubles using 20mL syringes

Order:

**Screw** on syringes

Turn nob to **JOG**

Press **RETRACT** button

Turn nob to **RUN**

Add **tick** mark to record progress

Wait for green **READY** light

Press **EXTEND** button

- Vehicle Control Mix – run 2x through and toss 12mL
- **Vehicle Control #1&2 (12mL)**
- **Vehicle Control #3&4 (12mL)**
- **Vehicle Control #5&6 (12mL)**
- Switch to 10mL syringes
- CBD #1 – run once through and toss
- **CBD #2**
- **CBD #3**
- Vehicle Control Mix – run 3x through and toss
- Extract #1 – run once through and toss
- **Extract #2**
- **Extract #3**

**Clean Up:**

- Reset pressure to 20kpsi
- Run with hot Dawn solution (we want to remove the oil first) until clear (not cloudy) blue
- Run with hot Micro90 solution until clear (not blue)
- Run 3x with room temperature MilliQ water (reduce detergent)
- Run with room temperature Zymit solution (to remove proteins) until clear
- Run with hot MilliQ water until clear
- Run 70% isopropanol until clear
- Turn off machine
