# Supplementary material for: Assessment of the effects of cannabidiol and a CBD-rich hemp extract in Caenorhabditis elegans
Source: Front Toxicol. 2024 Oct 1;6:1469341. doi: 10.3389/ftox.2024.1469341 (PMC11484448; doi:10.3389/ftox.2024.1469341)
Supplement: Supplementary file 3 [file Table3.docx]

**Modified wMicroTracker worm Development & Activity Test (wDAT+)**

| Strain: |  | Thaw Date: |  | CeHR prep date: |
| --- | --- | --- | --- | --- |
| **Day** | **Date** | **Temp.**  Room/Hood/inc. | **Room**  **Humidity** | **Notes:**  Dauers/Density/Culture Quality |
| P: feed |  |  |  |  |
| -1: egg prep |  |  |  |  |
| 0: Dose L1s |  |  |  |  |
| 1: Wash L2s |  |  |  |  |
| 2: L3-L4 |  |  |  |  |
| 3: L4-A |  |  |  |  |
| 4: progeny |  |  |  |  |

| Test Articles | Abbrev. | MW | CAS # | Notes |
| --- | --- | --- | --- | --- |
| Cannabidiol | CBD | 314.5 | 13956-29-1 |  |

|  | Dosing |  | Final | Waste |
| --- | --- | --- | --- | --- |
| L 0 | vehicle |  | 0 |  |
| L 1 | 3mg/mL | **CBD** | 300μg/mL |  |
| L 2 | 5mg/mL | **CBD** | 500μg/mL |  |
| L 3 | 7.5mg/mL | **CBD** | 750μg/mL |  |
| R 0 | vehicle |  | 0 |  |
| R 1 | 3mg/mL | **CBD eq. in extract** | 300μg/mL |  |
| R 2 | 5mg/mL | **CBD eq. in extract** | 500μg/mL |  |
| R 3 | 7.5mg/mL | **CBD eq. in extract** | 750μg/mL |  |

**Prepare dosing solutions**

- See Emulsion Worksheet

**wDAT+ Layout ex1**

| Col/  Row | 1 | 2 | 3 | 4 |  | 1 | 2 | 3 | 4 |
| --- | --- | --- | --- | --- | --- | --- | --- | --- | --- |
| A | **VCe** | **300 CBD** | **500 CBD** | **750 CBD** |  | **0.1% Tw80** | **VC** | **300 CBD** | **500 CBD** |
| B | **VCe** | **300 CBDeq** | **500 CBDeq** | **750 CBDeq** |  | **750 CBD** | **0.1% Tw80** | **VC** | **300 CBD** |
| C | **0.1% Tw80** | **0.1% Tw80** |  |  |  | **500 CBD** | **750 CBD** | **0.1% Tw80** | **VC** |

**Prep Day**

- Feed healthy N2 cultures

**Evening, Day -1**

- Put 0.1N NaCl, bleach, 5N NaOH, and M9 in hood
- Turn on centrifuge and set to 800 x g and 1 min
- Wipe down hood, centrifuge, frig and incubator handles with 70% isopropanol
- Update *C. elegans* maintenance table
- Worms in 0.1N NaCl *start time/hood temp* ________________
- Settle adult worms by gravity and wash in 0.1N NaCl 3x □ □ □
- N2 Egg Preps, *bleach open date _________, add bleach at __________*
- 1^st^ spin in hypochlorite  *time ____________*, 1^st^ spin in M9  *time ____________*
- Extra M9 washes □ □ (1 extra is enough if pellets are nearly dry)
- Egg Prep finished and in **M9** *time/hood temp* _________________+ 18h =
- **Transfer Parmalat from freezer to refrigerator for tomorrow!**

| **Prep:**  **Dauers:**  **Quality:**  **Count:** | **Prep:**  **Dauers:**  **Quality:**  **Count:** |
| --- | --- |

**Morning, Day 0**

**Preparation**

- Put water, milk (Parmalat), and CeHR in the incubator to warm up
- Wipe down hood, centrifuge, benches, handles with 70% isopropanol
- Assess hatched L1s from Egg Prep and update *C. elegans* maintenance table
- Set centrifuge to 2,500 x g for 5 min at 18°C
- Prepare dosing solutions (page 1)
- Centrifuge L1s and check pellet in scope
- Aspirate M9 supernatant from L1s, and resuspend in CeHM: *time/hood temp __________*
- Prepare at least 10mL of L1s in CeHM(parm) to ~3,000 worms/mL (10mL=30k)
- Prepare 30mL of ~1,000 worms/mL (30mL=30k)

**Notes on L1 worms in CeHM:**

Count: ___­­­­­­­­­­­­­­­­­__________­­­­­__________________ worms/10μL = _____ worms/μL

Count: ___­­­­­­­­­­­­­­­­­__________­­­­­__________________ worms/10μL = _____ worms/μL

Count: ___­­­­­­­­­­­­­­­­­__________­­­­­__________________ worms/10μL = _____ worms/μL

**Dosing**

- Add 900μL of ~3k worms/mL in CeHM to wDAT+ pattern plates
- Add 900μL of ~1k worms/mL in CeHM to wDAT pattern plates
- Add water to empty wells and spaces between wells
- Add 100μL of water or 10x dosing solution to wells *hood temp* __________
- Put wDAT+ plate into bottom left wMT and start evaluation *time ____________*
- Put wDAT plates into top wMTs and start evaluation *time ____________*
- If needed, transfer Parmalat from freezer to refrigerator for tomorrow

**Day 0**

- *Worms should be midL1 at about 7-10hpL1f, the L1 to L2 molt at about 18-21h*

**Day 1**

- *The L2 to L3 molt should occur at about 30-34h, L3 peak at about 35-40h*

**Day 1, wDAT+ only**

- Put water, milk (Parmalat), and CeHR in the incubator to warm up
- Wipe down hood, centrifuge, benches, handles with 70% isopropanol
- Turn on centrifuge and set to 800 x g and 1 min
- Label eight 15mL conical tubes for wDAT+ worm washing
- Prep two 50mL conicals to receive waste washes
- Label two 12-well plates for washed wDAT+ worms
- Prepare 30mL of CeHM(parm)
- At 24h post-dosing, stop and save wDAT+ wMT file only *time ____________*
- Microscopy Notes:

| Group | CBD | wDAT+ Notes at 24hpL1f + Dosing |
| --- | --- | --- |
| L 0 | vehicle |  |
| L 1 | 3mg/mL |  |
| L 2 | 5mg/mL |  |
| L 3 | 7.5mg/mL |  |
| Group | **CBD in Extract** |  |
| R 0 | vehicle |  |
| R 1 | 3mg/mL |  |
| R 2 | 5mg/mL |  |
| R 3 | 7.5mg/mL |  |

- For speed, remove plates and wash 2 wells of worms at a time
- Aspirate control washes directly into regular waste
- Carefully transfer waste supernatants to waste conicals
- Right plate hi wells: water □ M9 □ in CeHM *time* ____________
- Right plate lo wells: water □ M9 □ in CeHM *time* ____________
- Left plate hi wells: water □ M9 □ in CeHM *time* ____________
- Left plate lo wells: water □ M9 □ in CeHM *time* ____________
- Resuspend washed worms into 3mL CeHM and transfer to fresh 12-well plates
- Restart wDAT+ wMT recording *time ____________*

**Day 2**

- *The control L3 to L4 molt should occur around 40-44h, and they should be at mid-L4 at about 47-50h*

**Day 3**

- *The control L4 to A molt should occur around 52-57h, and they should be at A plateau by about 65h*

**Notes**

- Check curves, continue runs if exposed L4 data is not complete
- Save wDAT+ wMT data files *time ____________*
- Transfer wMT data files to F drive
- Microscopy Notes wDAT:

| Group | CBD | wDAT Notes at 3dpL1f & dosing |
| --- | --- | --- |
| L 0 | vehicle |  |
| L 1 | 3mg/mL |  |
| L 2 | 5mg/mL |  |
| L 3 | 7.5mg/mL |  |
| Group | **CBD in Extract** |  |
| R 0 | vehicle |  |
| R 1 | 3mg/mL |  |
| R 2 | 5mg/mL |  |
| R 3 | 7.5mg/mL |  |

- Save regular wDAT wMT data files *time ____________*
- Transfer wMT data files to F drive
- Microscopy Notes wDAT+:

| Group | CBD | wDAT+ Notes at 3dpL1f & 2 days post washing |
| --- | --- | --- |
| L 0 | vehicle |  |
| L 1 | 3mg/mL |  |
| L 2 | 5mg/mL |  |
| L 3 | 7.5mg/mL |  |
| Group | **CBD in Extract** |  |
| R 0 | vehicle |  |
| R 1 | 3mg/mL |  |
| R 2 | 5mg/mL |  |
| R 3 | 7.5mg/mL |  |
